# TEAD4 as an Oncogene and a Mitochondrial Modulator

**DOI:** 10.3389/fcell.2022.890419

**Published:** 2022-05-05

**Authors:** Sheng-Chieh Hsu, Ching-Yu Lin, Yen-Yi Lin, Colin C. Collins, Chia-Lin Chen, Hsing-Jien Kung

**Affiliations:** ^1^ Program for Cancer Biology and Drug Discovery, College of Medical Science and Technology, Taipei Medical University, Taipei, Taiwan; ^2^ Vancouver Prostate Centre and Department of Urologic Sciences, University of British Columbia, Vancouver, BC, Canada; ^3^ Research Center of Cancer Translational Medicine, Taipei Medical University, Taipei, Taiwan; ^4^ Institute of Molecular and Genomic Medicine, National Health Research Institutes, Zhunan, Taiwan; ^5^ Department of Biochemistry and Molecular Medicine, Comprehensive Cancer Center, University of California, Davis, Sacramento, CA, United States

**Keywords:** mitochondria, oxphos, epigenetics, Tead4, cancer

## Abstract

TEAD4 (TEA Domain Transcription Factor 4) is well recognized as the DNA-anchor protein of YAP transcription complex, which is modulated by Hippo, a highly conserved pathway in Metazoa that controls organ size through regulating cell proliferation and apoptosis. To acquire full transcriptional activity, TEAD4 requires co-activator, YAP (Yes-associated protein) or its homolog TAZ (transcriptional coactivator with PDZ-binding motif) the signaling hub that relays the extracellular stimuli to the transcription of target genes. Growing evidence suggests that TEAD4 also exerts its function in a YAP-independent manner through other signal pathways. Although TEAD4 plays an essential role in determining that differentiation fate of the blastocyst, it also promotes tumorigenesis by enhancing metastasis, cancer stemness, and drug resistance. Upregulation of TEAD4 has been reported in several cancers, including colon cancer, gastric cancer, breast cancer, and prostate cancer and serves as a valuable prognostic marker. Recent studies show that TEAD4, but not other members of the TEAD family, engages in regulating mitochondrial dynamics and cell metabolism by modulating the expression of mitochondrial- and nuclear-encoded electron transport chain genes. TEAD4’s functions including oncogenic activities are tightly controlled by its subcellular localization. As a predominantly nuclear protein, its cytoplasmic translocation is triggered by several signals, such as osmotic stress, cell confluency, and arginine availability. Intriguingly, TEAD4 is also localized in mitochondria, although the translocation mechanism remains unclear. In this report, we describe the current understanding of TEAD4 as an oncogene, epigenetic regulator and mitochondrial modulator. The contributing mechanisms will be discussed.

## Introduction

**FIGURE 1:**
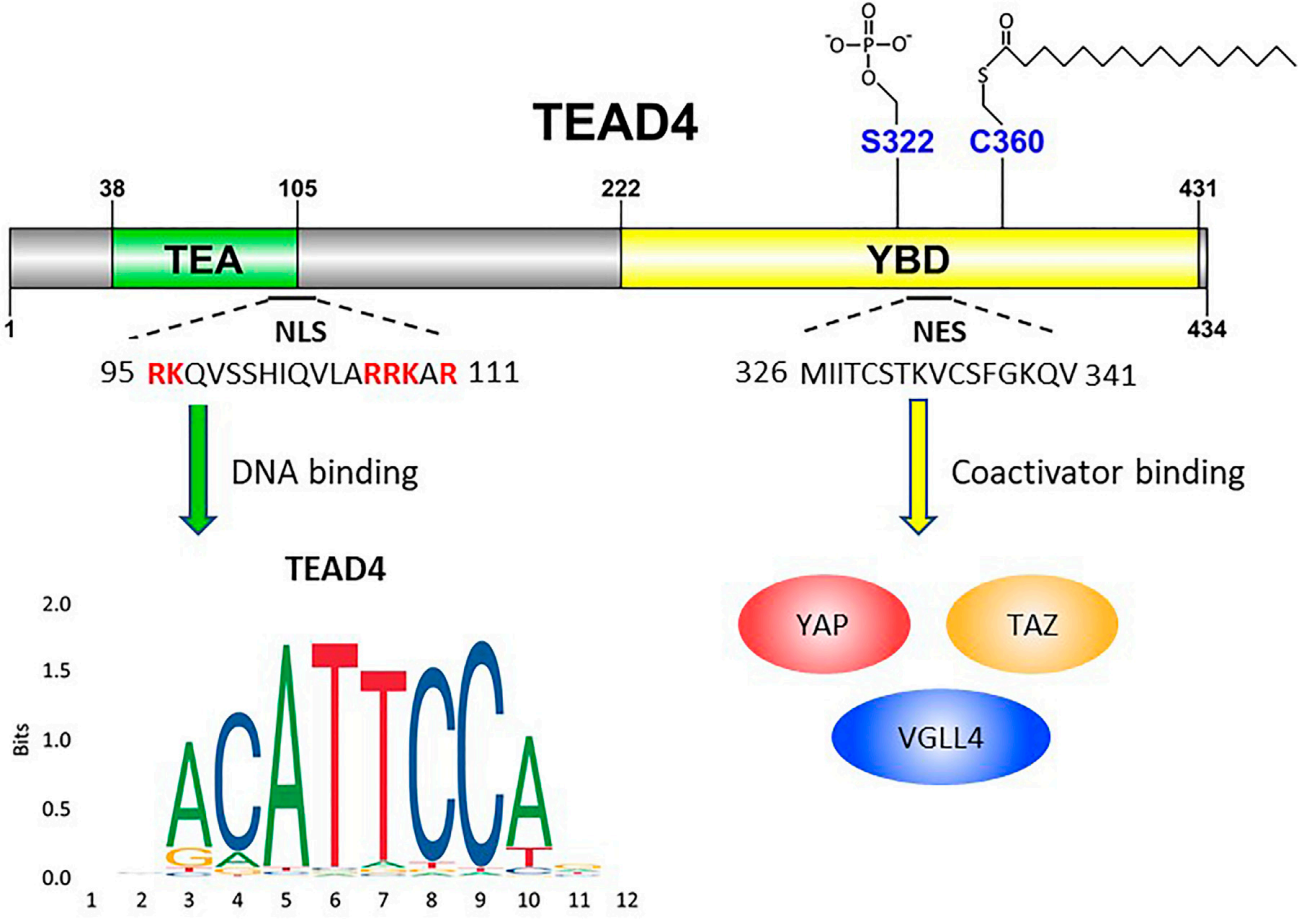
Schematic illustration of TEAD4’s functional domains. TEA domain (38–105) is named after TEF-1 and abaA, both of which contain this domain for DNA binding. The basic amino acids (highlighted in red) are crucial for TEAD4 nuclear translocation. YBD, YAP-binding domain (222–431). S322 is the serine residue for phosphorylation. C360 is the cysteine residue for palmitoylation. YAP, TAZ and VGLL4 are transcription factors which bind YAP domain.

TEAD4 belongs to a family of TEA (Transcriptional Enhanced Associate) domain containing transcription factors which include TEAD1, 2, 3 and 4. They share significant homology and common functions, but also unique properties (Huh et al., 2019). TEAD4 carries a TEA DNA binding domain near the N-terminus and a YBD (YAP-binding domain) at the C-terminus. The DNA binding domain is highly conserved in four TEAD members and binds to the MCAT element (5′- CATTCCA/T-3′) of DNA (Hwang et al., 1993; Jiang et al., 2000) (Figure 1). However, the DNA binding domain lacks a transactivation domain and generally requires a coactivator to exert its transcription functions. In contrast, the consensus sequence of YBD is more diverse across four TEAD members, suggesting that it might have different three-dimensional structures with differential affinity toward co-activators for transcriptional regulation. By interacting with these co-activators, TEAD4 modulates the expression of distinct gene sets to affect different biological and disease processes TEAD4 plays a notable role distinct from other three TEADs in both embryonic development as well as tumorigenesis (Chen et al., 2020). A major partner of TEAD4 in these processes is YAP (Yes-associated protein) or its homolog TAZ (transcriptional coactivator with PDZ-binding motif, also known as WW domain containing transcription regulator 1), which forms a transcription complex with TEAD4 to reprogram the transcriptome. The YAP/TEAD4 axis is negatively regulated by Hippo pathway which responds to microenvironmental factors, such as cell adhesion, cell-cell contact, or stress signal (Moya and Halder, 2019). Hippo pathway acts as a gatekeeper of cell growth, loss of Hippo pathway or overexpression of YAP/TEADs is repeatedly observed in various cancers (Zanconato et al., 2016). Recent studies showed that TEAD4 can also partner with other coactivators to direct transcription in a YAP-independent manner (Lin et al., 2017b). Furthermore, TEAD4 has multiple cellular locations including nucleus, cytosol and mitochondria (Kaneko and DePamphilis, 2013) with a new found role in transcriptional activation of OXPHOS genes in both nucleus and mitochondria (Kumar et al., 2018; Chen et al., 2021). In this review, we shall discuss TEAD4 as a mitochondrial modulator and an oncogene, characterized by it transcriptional potential as an epigenetic regulator and a signal transducer.

### TEAD4 as an Epigenetic Regulator of Transcription

One striking feature of TEAD4 as a transcription factor is its binding is closely aligned with “open chromatin” or “super enhancer” as exemplified by the decoration of acetylated histone H3K27ac. Several coactivators for TEAD4 have been identified (Vassilev et al., 2001; Mahoney et al., 2005; Liu X. et al., 2016) and see below), among which the most recognized is YAP and TAZ (Totaro et al., 2018). TEAD4 recruits YAP/TAZ to their DNA binding loci (Zanconato et al., 2015). Based on ENCODE ChIP-Seq database, more than 75% of YAP/TAZ binding sites comprise the TEAD4 binding sequence, and about 78% of YAP/TAZ peaks are colocalized with TEAD4 peaks. Although TEAD4 regulates gene expression as a transcription factor, only a small fraction of its binding sites is located in the promoter regions. Accumulating evidence shows that most TEAD4 peaks reside in enhancers, which is farther than 10 kb of the nearest transcription start site and identified by the bimodal distribution of H3K4me1 around its peaks. The enhancer-enriched property is universal, since this pattern has been observed across several cancer cell lines. These TEAD4-enriched enhancers are transcriptionally active due to the co-occupancy of H3K27ac in a YAP/TAZ dependent manner (Zanconato et al., 2015). In a detailed study of TEAD4’s association with open chromatin, Zemke et al. used ATAC-seq, an approach to identify chromatin accessibility and reported that the ATAC signals greatly overlapped with H3K27ac super-enhancer peaks, which were highly enriched with YAP/TEAD4 occupancy (Zemke et al., 2019). At the same time, H3K4me1 peaks were shifted away from the center of ATAC-sites, indicating that chromatin undergoes remodeling following YAP and TEAD4 binding. These results suggest that TEAD4 is involved in the *de novo* formation of super-enhancers, perhaps by creating nucleosome-depleted regions for the association of other transcriptional factors (Zemke et al., 2019). TEAD4 also interacts with AP-1 (Activator protein 1) to regulate gene expression (Liu X. et al., 2016; Obier et al., 2016). AP-1 motif is found nearby the TEAD4 motif of most YAP/TAZ/TEAD4 peaks. The ChIP-seq result shows that 78% of YAP/TAZ/TEAD4-binding sites are co-occupied by JUN, a subunit of AP-1, and more than 90% of the co-occupancies belong to active enhancers. Similarly, two other AP-1 factors, FRA1/2 (Fos-related antigen 1/2), also interact with TEAD4, and ChIP-seq data showed that TEAD4 binding sequence is the most enriched motif in FRA1/2 peaks, indicating a strong association between AP-1- and TEAD4-mediated transcriptional module (Liu X. et al., 2016). The interaction between TEAD4 and AP-1 is independent of JNK (c-Jun N-terminal Kinase), the upstream kinase of AP-1; but requires SRC (steroid hormone receptor co-activator). The TEAD4/AP-1/SRC complex modulates a set of genes, including DOCKs (Dedicator of cytokinesis), CDH2 (Cadherin 2), and MACF1(Microtubule Actin Crosslinking Factor 1), to regulate cancer metastasis. TEAD4 also cooperates with SMAD3 (SMAD Family Member 3) to regulate epithelial-to-mesenchymal transition (EMT) by activating mesenchymal-specific genes under TGFβ (Transforming growth factor beta) stimulation. In TGFβ-induced EMT, TEAD4 and SMAD3 co-occupy the enhancers of SNAI2 (Snail Family Transcriptional Repressor 2) and ITGB3 (Integrin Subunit Beta 3) (Qiao et al., 2020). Depletion of TEAD4 not only decreases its binding to mesenchymal-specific enhancers but also significantly reduces H3K27ac on these enhancers. This result shows that TEAD4-SMAD3 complex promoting activation of the mesenchymal enhancers by upregulating H3K27ac acquisition. Recently it was further shown TEAD4 is coupled to RAD51 (RAD51 Recombinase) to form “oncogenic super enhancers” which couples DNA repair to hyper activation of oncogenes (Hazan et al., 2019). These data together provide strong evidence that TEAD4 plays an important role in shaping the “super enhancers” by histone acetylation and potently activating the target genes.

In addition to promoting gene expression through enhancing H3K27ac, TEAD4 also suppresses gene expression by recruiting EZH2 (Enhancer of Zeste 2 Polycomb Repressive Complex 2 Subunit) to promoters and are crucial for the maintenance of trophoblast stemness (Meinhardt et al., 2020). During embryonic development, TEAD4 plays a key role in determining the cell fate of the villous cytotrophoblasts (vCTBs) (Haider et al., 2016; Soncin et al., 2018). Together with YAP, TEAD4 promotes cell proliferation by upregulating CCNA1 (Cyclin A1) and CDK6 (Cyclin Dependent Kinase 6) through binding to their promoter and enhancer respectively. Meanwhile TEAD4-YAP complex also interacts with EZH2 resulting in the upregulation of repressive histone mark H3K27me3 in the promoters of CGB5 (Chorionic Gonadotropin Subunit Beta 5) and CGB7, syncytiotrophoblast (STB)-specific genes, leading to suppression of STBs differentiation (Meinhardt et al., 2020).

### TEAD4 as a Transducer of Upstream Signals

**FIGURE 2:**
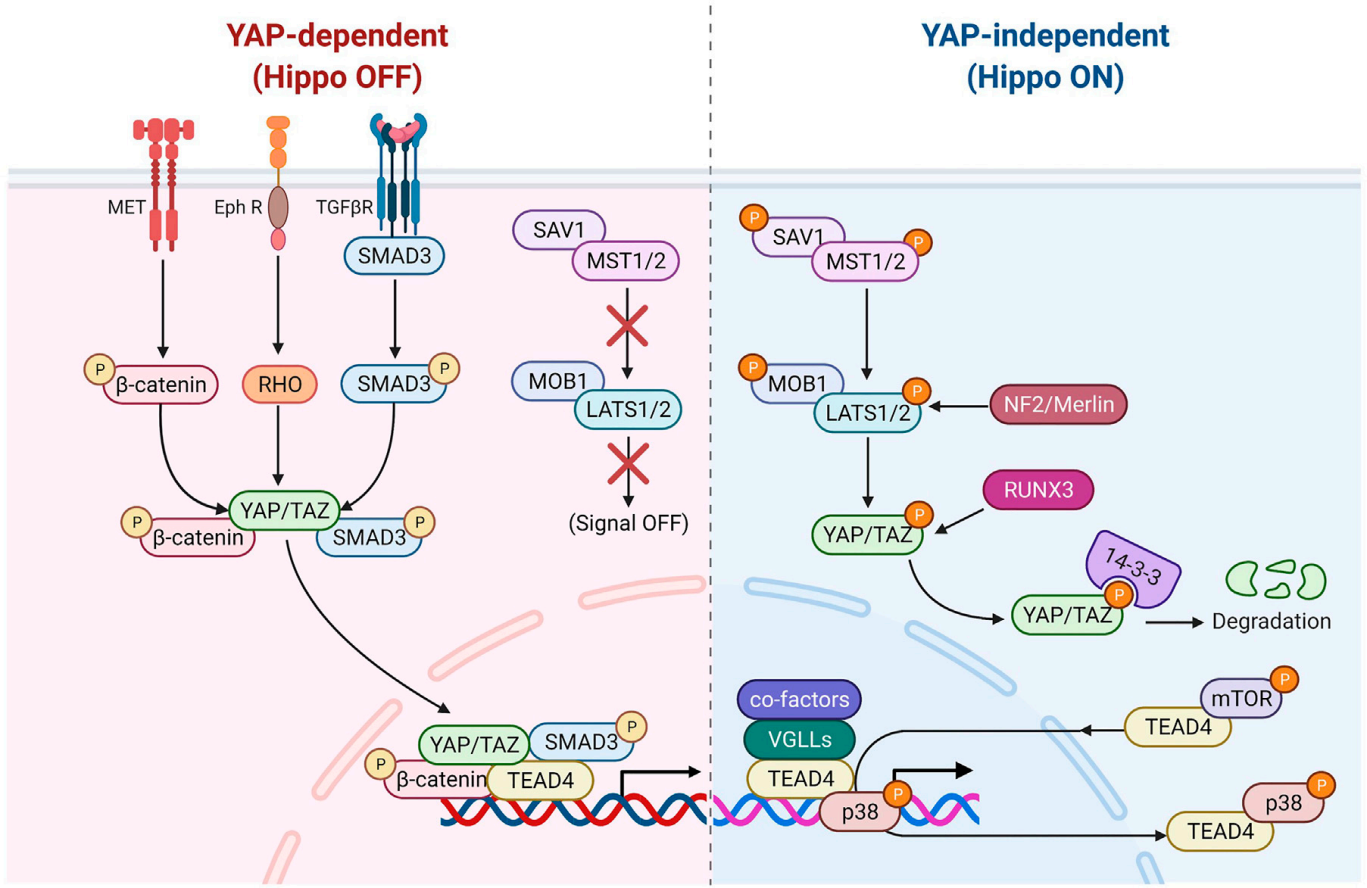
TEAD4 upstream signals (YAP-dependent). Once the Hippo pathway is inactivated, the nuclear translocation of YAP can be modulated by several signaling pathways, including HGF induced β-catenin pathway, TGFβ-induced SMAD pathway and Ephrin A2 induced Rho-dependent pathway. Several antiviral associated pathways also can modulate YAP nuclear translocation or its binding activity of TEAD4.

**FIGURE 3:**
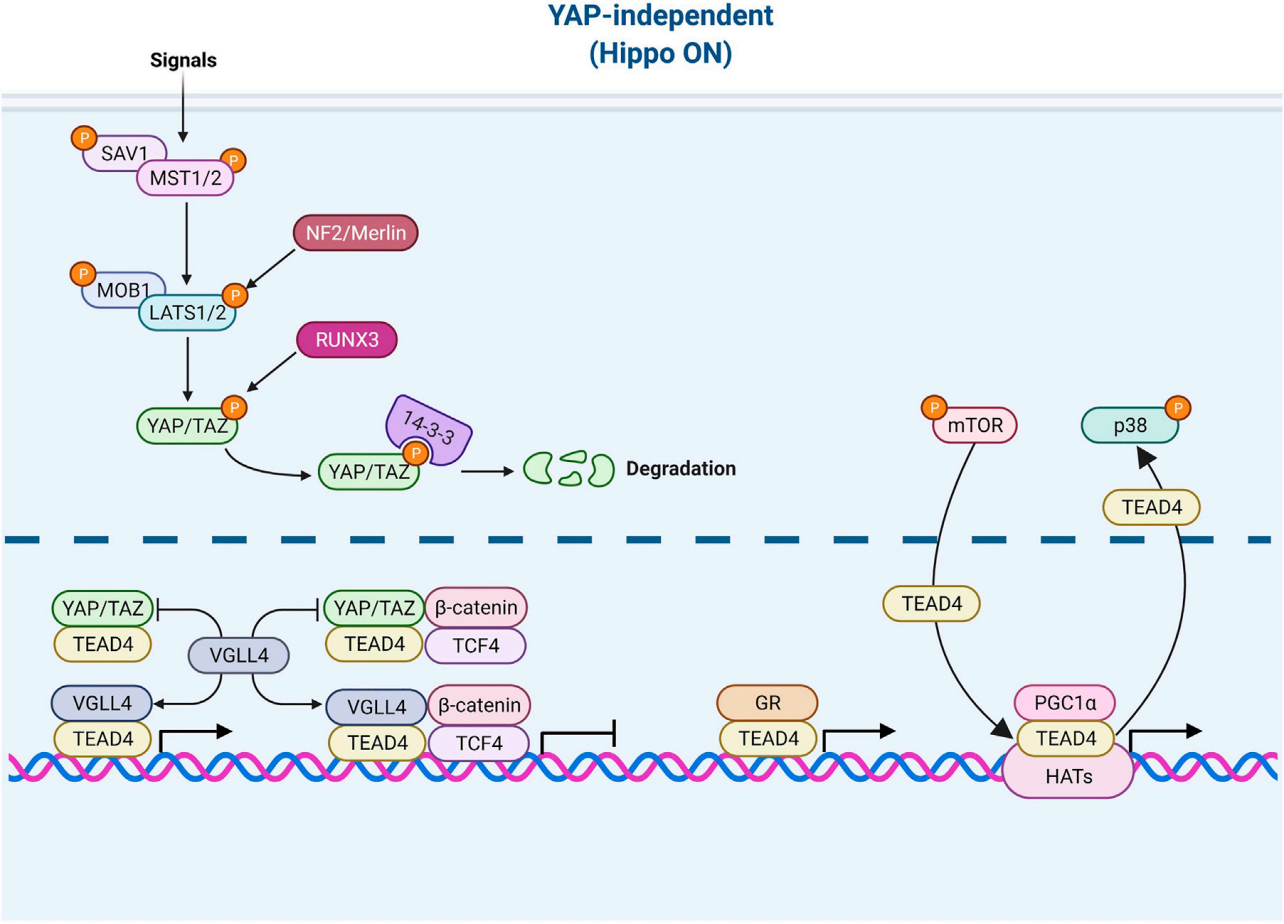
TEAD4 upstream signals (YAP-independent). Once the Hippo pathway is activated by signals (such as environmental stress or extracellular contacts), YAP is phosphorylated and restrained in cytosol. VGLL4 can act as YAP antagonist and TEAD4 coactivator as well. The nuclear glucocorticoid receptor (GR) and PGC1α can directly bind to TEAD4 in a YAP-independent manner. mTOR and p38 signaling are involved in nuclear retention and cytosolic translocation of TEAD4.

Being a DNA anchor protein, TEAD4’s ability to reprogram transcription depends on upstream signals transmitted through coactivators which bind TEAD4. They can be generally categorized as YAP-dependent (Figure 2) and YAP-independent (Figure 3). There are also other signals which regulate the subcellular locations and functions of TEAD4.

#### YAP-Dependent (Hippo-Off)

As described above, YAP is considered as a major co-activator of TEADs family including TEAD4 and their associations are firmly established in several systems (Cao et al., 2008; Chen et al., 2010; Hau et al., 2013; Kaan et al., 2017; Mesrouze et al., 2017; Shi et al., 2017; Li et al., 2018; Niu et al., 2019). During the development of mouse trophectoderm (TE), Hippo pathway determines the cellular location of YAP (Nishioka et al., 2009). Unlike outer cell mass where YAP translocates to nuclei and integrates with TEAD4 to promote TE differentiation (Hippo-off), in inner cells, Hippo pathway is activated and YAP is phosphorylated by Large Tumor Suppressor kinases 1 and 2 (LATS1/2) and retained in cytosol, diminishing TEAD4 functions (Nishioka et al., 2009; Hirate et al., 2012; Meinhardt et al., 2020). This lineage specification is determined by NF2 (Neurofibromatosis 2)/Merlin (gene product of NF2)-LATS1/2 axis (Cockburn et al., 2013). Another study showed that LATS1/2 mediates the phosphorylation of YAP and its interaction with the tumor suppressor RUNX3 (RUNX Family Transcription Factor 3), which leads to dissociation of YAP/TEAD4 in the eye cell fate determination (Jang et al., 2017). Frum et al. showed that YAP/TAZ/TEAD4 suppresses Sox2 (SRY-Box Transcription Factor 2) expression at the 4 to 8-cell stage and then the Hippo pathway is activated at 16-cell stage, leading to phosphorylation of YAP/TAZ and dissociation from TEAD4 (Frum et al., 2019).

In addition to stress signals, YAP/TEAD4 also receives signals from membrane receptors including TGFβR, EPHR (Ephrin receptor) and MET (MET Proto-Oncogene, Receptor Tyrosine Kinase). TGFβ activates SMAD3 to form YAP/TEAD4/SMAD3/p300 complex in regulating the expression of connective tissue growth factor (CTGF) in mesothelioma (Fujii et al., 2012). Receptor tyrosine kinase EPHR activated in breast cancer upregulates YAP/TAZ expression and increases YAP accumulation and the expression of YAP/TEAD4 target genes (Edwards et al., 2017). The tyrosine protein kinase, MET, induces phosphorylation of β-catenin which binds and translocates YAP into nucleus to form a transcriptional complex with TEAD4 in breast cancer (Quinn et al., 2021).

Interestingly, several anti-viral signaling pathways modulate the YAP/TEAD4 activity. Jiao et al. discovered that the virus infection (Sendai virus, Vesicular Stomatitis Virus, and Hepatitis C virus)-induced antiviral signaling (such as RIG-I-MAVS and STING-cGAS pathways) suppresses the phosphorylation of YAP and promotes its nuclear translocation. Moreover, these virus-induced signals increase the expression of IRF3 (Interferon Regulatory Factor 3), a key regulator of innate immunity, which binds to YAP/TEAD4 complex and upregulates YAP target genes in gastric cancer (Jiao et al., 2018). Luo et al. uncovered HBV infection induces Toll-like receptor signaling that suppresses Hippo pathway *via* TLR2-MYD88-URAK4 axis, leading to YAP nuclear translocation to engage TEAD4 in hepatocytes (Luo et al., 2021).

#### YAP-Independent (Hippo-On)

While the major coactivator of TEAD4 is YAP, TEAD4 also functions when Hippo pathway is active which accelerates the degradation of YAP. For instance, various members of VGLL (the vestigial-like protein) were found to directly interact with TEAD4 in the absence of YAP (Mesrouze et al., 2014; Gao et al., 2019). In fact, VGLL was characterized as a YAP antagonist and a tumor suppressor (Jiao et al., 2014). VGLL4 interrupts YAP/TEAD4 complex in liver cancer (Feng et al., 2020) and TEAD/TCF4 (Transcription Factor 4) complex to suppress Wnt/β-catenin downstream targets (Jiao et al., 2017). Zhang et al. further showed that TEAD4 suppresses adipogenesis by recruiting VGLL4 and CtBP2 (C-Terminal Binding Protein 2) (Zhang et al., 2018b). VGLL4 has a dual role in muscle regeneration. At early stage, VGLL4 acts as an antagonist of YAP to induce myoblast proliferation. Later, VGLL4 acts as a coactivator of TEAD4 and form a transcription complex with MyoD (Myoblast Determination Protein 1) to promote myoblast differentiation (Feng et al., 2019). Figeac et al. found that another VGLL member, VGLL3, also can regulate myogenesis *via* interacting with TEAD1, 3 and 4 (Figeac et al., 2019). Another coactivator of TEAD4 is glucocorticoid receptor which binds TEAD4 and acts, in a YAP-independent manner, to promote transcription in breast cancer (He et al., 2019; Park et al., 2019). A recent study revealed that TEAD4 activates nuclear-encoded mitochondrial OXPHOS genes by interacting with PGC-1α (peroxisome proliferator-activated receptor gamma coactivator 1-alpha), a coactivator involved in mitochondrial biogenesis. YAP was absent from the targeting complex and its knockdown had no bearing on OXPHOS (Kumar et al., 2018; Chen et al., 2021). Under arginine replete condition, mTOR signaling pathway induces the recruitment of TEAD4 and histone acetyltransferases to the promoters of OXPHOS genes, pointing to the kinship of TEAD4 and histone acetylation (Chen et al., 2021).

#### Other Regulatory Signals

TEAD4 can be regulated by the interactions with coactivators, but also by its subcellular locations. TEAD4 is primarily a nuclear protein and exerts its transcriptional functions in the nucleus. There are however signals which regulate its nuclear-cytosolic translocation. TEAD4 protein contains both a highly conserved nuclear localization sequence (NLS) and nuclear export sequence (NES) (Figure1). In *Drosophila*, it was shown that the conserved bipartite NLS in the N-terminus of Sd (TEAD4 ortholog), and the six basic amino acids in the NLS are mandatory for Sd nuclear translocation by importin-α3 (Magico and Bell, 2011). In HUVEC cells, the putative NLS of TEAD4 was mapped to the N-terminal L105 to K109 (Liu et al., 2011), deletion of which abolished its ability to activate VEGF induced angiogenesis. Although TEAD4 cytoplasmic translocation is not observed very often in routine cell culture, it has been reported in the development of embryo, and the nuclear localization, not the expression, of TEAD4 determines the cell lineage commitment (Home et al., 2012). TEAD4 nuclear-cytoplasmic translocation is triggered by several stimuli such as cytokine and environmental stress. In human ESCs, BMP4 (Bone Morphogenetic Protein 4) treatment causes nuclear translocation of TEAD4 and activates TEAD4 target gene such as GATA3 (GATA-binding protein 3) (Home et al., 2012). Hyperosmolality, overconfluent cell culture, cell detachment, and arginine deprivation also induce TEAD4 nuclear export (Lin et al., 2017a; Chen et al., 2021). One underlying mechanism could be the activation of stress induced kinase p38 MAPK which triggers TEAD4 nuclear export (Lin et al., 2017a). Both TEAD4-p38 interaction and p38 kinase activity are required for hyperosmolality-induced TEAD4 nuclear export, but direct phosphorylation by p38 was not observed. The interaction is mediated directly through the TEA DNA binding domain of TEAD4, which overlaps with NLS. p38 thus may disrupt TEAD4-DNA interaction leading to the expulsion of TEAD4 from its target sites and nucleus.

The functions of transcription factors are often modulated by posttranslational modifications (PTM). The serine residue 322 (S322) on YBD was identified as a phosphorylation site; the upstream kinase, however, remains unclear (Ueyama et al., 2000) (Figure 1). The most well-studied PTM of TEADs is palmitoylation. The conserved cysteine residues (C344, C380, C371 and C360 for TEAD1 to 4 respectively) of TEAD members for palmitoylation were identified by two different groups (Chan et al., 2016; Noland et al., 2016). Palmitoylation is important for protein folding and stability of TEADs (Noland et al., 2016) and critical for the interaction of TEADs with its coactivators, including YAP and VGLL4. This process is regulated by the abundance of palmitate and its synthesizing enzyme, FASN (fatty acid synthase), which is in turn negatively regulated by NF2 (Merlin) (Kim and Gumbiner, 2019). It is noteworthy that TEAD4, not TEAD1, is particularly sensitive to cell-cell contact and silencing of FASN. Since palmitoylation is so crucial to TEAD4’s activity, its inhibitor currently evaluated for cancer therapy represents another option (Li et al., 2018).

### TEAD4 as a Mitochondrial Modulator

TEAD4, but not other members of this family, has another novel property of modulating the expression of both nuclear and mitochondrial-encoded OXPHOS (oxidative phosphorylation) genes. Kaneko and DePamphilis first reported the unique function of TEAD4 in mitochondrial homeostasis (Kaneko and DePamphilis, 2013). They showed that Tead4 is localized in mitochondria, in addition to nucleus. Knocking out Tead4 in mouse embryonic fibroblasts reduced mitochondrial membrane potential and increased ROS (reactive oxygen species) production. Subsequently, Kumar et al. showed that in mouse trophoblast stem cells, Tead4 was translocated to mitochondria and in those cells, mitochondria were more elongated with increased cristae and more active (Kumar et al., 2018). Silencing of TEAD4 not only suppressed cristae formation but also caused mitochondrial dysfunction, such as reduction in OXPHOS, loss of mitochondrial membrane potential, and surge in mitochondrial ROS (Kumar et al., 2018). TEAD4 promotes the expression of mtDNA (mitochondrial DNA)-encoded OXPHOS genes by increasing mitochondrial RNA polymerase (POLRMT) binding to mtDNA. TEAD4 recognizes the consensus TEA motif on mtDNA and interacts with POLRMT to drive mtDNA transcription (Kumar et al., 2018). It is noteworthy that knocking down of YAP does not alter the mtDNA transcription, indicating that TEAD4 regulates mtDNA transcription in a YAP-independent manner. In addition, we showed that TEAD4 is also a master regulator of nuclear-encoded OXPHOS genes, and its function is induced by amino acid arginine (Chen et al., 2021).

**FIGURE 4:**
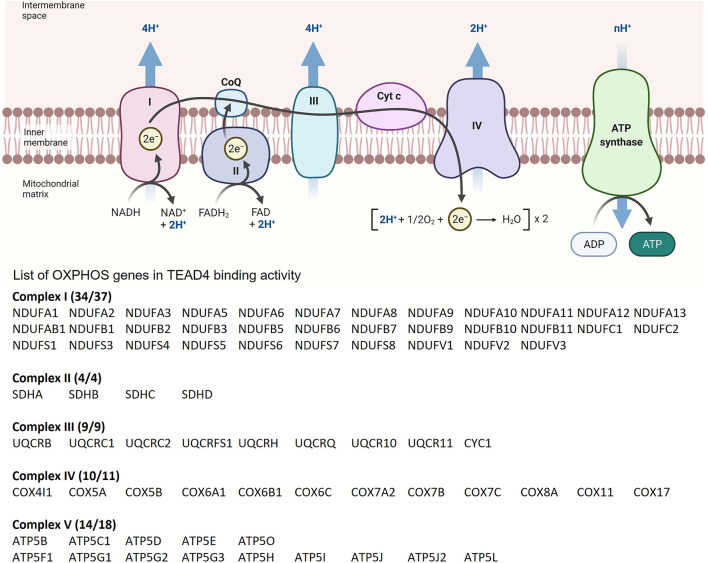
List of OXPHOS loci which contain TEAD4 peaks based on ChIP-seq results from the ENCODE Transcription Factors Targets dataset.

Arginine induces the chromatin remodeling *via* histone acetylation, which results in TEAD4 recruitment to TEA motifs present in >46% of the promoter/enhancer of OXPHOS genes with consequent gene activation and enhanced mitochondrial respiration. Significantly, ENCODE ChIP-seq dataset shows that 90% of OXPHOS genes (71/79) are direct targets of TEAD4 (i.e., with TEAD4 occupancy at the promoter/enhancer), implicating TEAD4 as a key regulator of OXPHOS genes (Figure 4). We also found that TEAD4-driven transcription of nuclear encoded OXPHOS genes is YAP-independent and PGC-1αdependent. The mitochondrial OXPHOS complexes consists of 88 proteins, 13 encoded by mitochondrial genome and the rest by nuclear genome. It is thus remarkable that TEAD4 is able to coordinately regulate the transcription of both mitochondrial and nuclear-encoded OXPHOS genes with profound impact on mitochondrial activities.

### TEAD4 as an Oncogene

**FIGURE 5:**
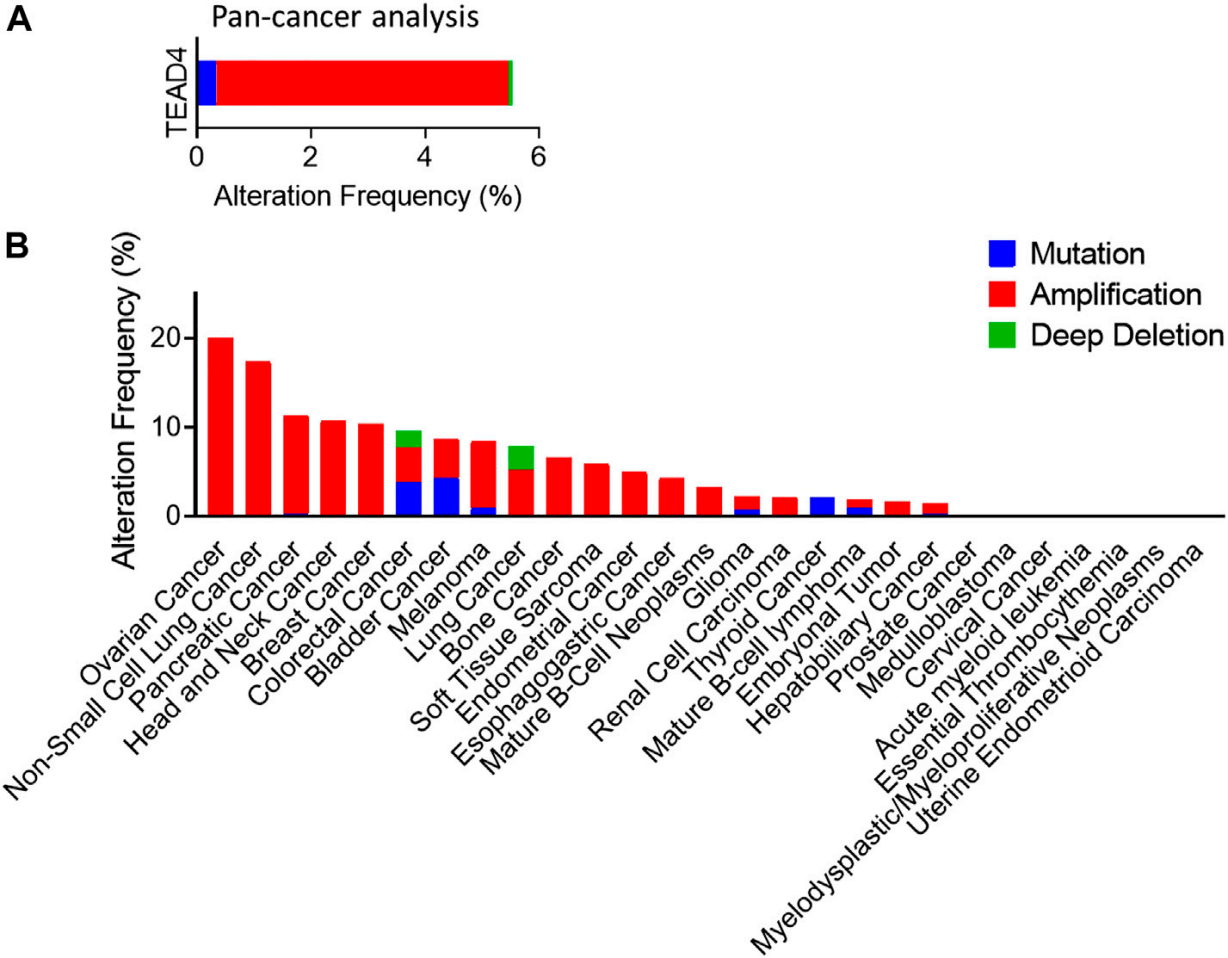
Transcriptomic profiles of TEAD4 in various cancers. **(A)** In the Pan-Cancer analysis (ICGC/TCGA, Nature 2020), 6% of samples possess TEAD4 abnormality. **(B)** Most common alteration is gene amplification found in ovarian cancer, non-small cell lung cancer, and pancreatic cancer. In ovarian cancer, 20% of tumors contain TEAD4 amplification.

There is considerable evidence implicating TEAD family transcription factors in cancer development. Copy number amplification, single nucleotide polymorphism (SNP) and overexpression of TEADs are observed in many cancers with clinical relevance (Table 1). Among the four TEAD members, the elevated TEAD4 expression is the most frequently observed in various types of cancers and highly associated with clinical significance, such as cancer progression or patient’s survival rate. Based on Pan-Cancer analysis of whole genomes, 6% of 2,565 cancer patients analyzed in that study possess TEAD4 abnormality (ICGC/TCGA Pan-Cancer Analysis of Whole Genomes Consortium, 2020). Gene amplification is the most common alteration in 30 different cancer types (Figure 5). For general discussion on the role of TEAD family in cancers, readers are referred to recent excellent reviews (Zhou et al., 2016; Huh et al., 2019). In this article, we will focus specifically on the unique roles of TEAD4 in oncogenesis. As described below, through the activation of different target genes, TEAD4 has the potential to induce proliferation, anti-apoptosis, EMT, migration, and metastasis, depending on the upstream signals and the coactivators associated with.

**TABLE 1:** TEADs overexpression and clinical relevance in various cancer types.

TEADs	Cancer Type	Clinical Association	Signals and Its Oncogenic Targets	References
TEAD1	Melanoma	Poor prognosis	-	Yuan et al. (2015)
	Prostate cancer	Lower survival rate	-	Knight et al. (2008)
	Renal cell carcinoma	Tumor growth, migration	YAP/TEAD1/CTGF/MYC/EDN1/EDN2	Schutte et al. (2014)
	Thyroid cancer	Tumorigenesis	YAP/TEAD1/Ras/MEK/ERK	Garcia-Rendueles et al. (2015)
TEAD2	Liver cancer	Lower survival rate and poor prognosis	TEAD2/VGLL4/EMT	Joo et al. (2020)
	Ovarian serous carcinoma	Lower survival rate and progression-free survival	-	Ren et al. (2021)
TEAD3	Liver cancer	Higher overall survival	-	Xia et al. (2017)
	Pancreatic Cancer	Poor prognosis	-	Hashimoto et al. (2019)
	Renal cell carcinoma	-	SAV↓/YAP1↑/TEAD3↑	Matsuura et al. (2011)
TEAD4	Bladder cancer	Induction of EMT, poor prognosis	TEAD4/CDH1/CDH2/FN1/TWIST1/2	Huang et al. (2021)
	Bladder cancer	Poor prognosis	-	Wang J. et al., 2021
	Breast cancer	-		Han et al. (2008)
	Breast cancer	Metastasis, recurrence	TEAD4/KLF5/p27	Wang C. et al., 2015
	Breast cancer	Poor prognosis	GR/TEAD4/CDH2/ANKRD1/BIRC5	He et al. (2019)
	Colorectal cancer	Metastasis, poor prognosis	TEAD4/SIX1/CDH1	Yu et al. (2021)
	Colorectal cancer	Metastasis, poor prognosis	TEAD4/CDH1/VIM	Liu X. et al., 2016
	Colorectal cancer	Poor prognosis	YAP/TEAD4/RANBP1	Zheng et al., 2022
	Esophageal Cancer	Tumor growth, migration, invasion	YAP1/TEAD4/SGK1/MMP2/MMP9	He et al. (2019)
	Gastric cancer	Poor prognosis	TEAD4/ADM/ANG/ARID5B/CALD1/EDN2/FSCN1/OSR2	Lim et al. (2014)
	Gliomas	Poor prognosis		Yuan et al. (2021)
	Head neck squamous cell carcinoma	Induction of EMT	TGFb/TEAD4/CDH1/CDH2/VIM/SNAI1	Zhang et al., 2018a; He et al. (2021)
	Liver cancer	Poor prognosis	YAP/TEAD4/Jag-1/Hes-1	Tschaharganeh et al. (2013)
	Liver cancer	Tumor growth	TEAD4/HSPA6/HSPA1A	Coto-Llerena et al. (2021)
	Lung cancer	Poor prognosis	TEAD4/PKM2/HIF1a	Hu et al., (2021)
	Melanoma	Poor prognosis	-	Yuan et al. (2015)
	Ovarian cancer	Poor prognosis	YAP/TEAD4/CDH1/SNAI1	Xia et al., 2014a
	Ovarian cancer	Metastasis, poor prognosis	TEAD4/RPS27A/RPS2	Ren et al. (2021)
	Ovarian cancer, fallopian tube carcinoma	-	-	Nowee et al. (2007)
	Prostate cancer	Lower survival rate and tumor recurrence	mTOR/TEAD4/KATS/OXPHOS	Chen et al. (2021)
	Renal cell carcinoma	Tumor grade and lower survival	-	Li et al. (2022)
	Thyroid cancer	Suppression of tumor progression and metastasis	TEAD4/Wnt3a/CDH1/CDH2/VIM	Zhang et al. (2022)
	Lung cancer, colon cancer, neuroblastoma, endometrial cancer	Metastasis	YAP/TEAD4/AP-1/SRC/CDH2/MACF1	Liu X. et al., 2016
	Lung cancer, liver cancer	Metastasis	TEAD4/SMAD3/SNAI2/ITGB3	Qiao et al. (2020)
TEAD1/4	Gastric cancer	Poor prognosis	miR-4269↓/miR-377-3p↓/miR-1343-3p↓/TEAD4↑	Zhou et al. (2017)
TEAD2/4	Gliomas	Lower survival rate	TAZ/TEAD2/mesenchymal genes	Bhat et al. (2011)
TEAD1/3/4	Ovarian cancer	Chemoresistance	YAP/TEADs/GSK3A/ABCB1	Xia et al., 2014b
TEAD1/2/3/4	Esophageal Cancer	Tumor progression	YAP/TEADs/JNK/c-Jun/IRS2	Xu et al. (2021)

#### Liver Cancer

The Hippo pathway plays a vital role in the normal functions in liver, including metabolic homeostasis, cell proliferation and regeneration. It goes without saying that the deviant regulations of Hippo signaling are frequently observed in liver cancer (Nguyen-Lefebvre et al., 2021). In human hepatoma cells, TEAD4 exerts the antiapoptotic activity by upregulating Jag-1 expression, a Notch ligand (Tschaharganeh et al., 2013). By competing out HNF4a, a tumor suppressor involved in hepatocyte differentiation, YAP forms complex with TEAD4 to up-regulate genes associated with cell proliferation (Cai et al., 2017). YAP/TEAD4 also binds FOXM1 (Forkhead Box M1) to induce the chromosome instability (Weiler et al., 2017) and targets PAI-1 (plasminogen activator inhibitor-1) to control the senescence in liver cancer (Marquard et al., 2020). Thomann et al. further showed that YAP/TEAD4 induces osteopontin, which stimulates c-Met expression and alters the tumor niche (Thomann et al., 2020). It also induced a macrophage chemoattractant CCL2 (C-C Motif Chemokine Ligand 2) expression to modulate the innate immunity and tumor microenvironment (Thomann et al., 2021). It is worth mentioning that TEAD4 also can modulate the liver cancer progression in a YAP-independent manner. Upon TGFβ stimulation, TEAD4 can form a complex with SMAD 2/3 on mesenchymal-specific enhancers, resulting in EMT process in liver and lung cancers (Qiao et al., 2020). In a YAP-independent manner, TEAD4 induces the expression of 70-kDa heat shock protein (HSP70) family members, a key driver for liver tumor growth (Coto-Llerena et al., 2021). In liver cancer, TEAD4 is not the only TEAD member implicated in oncogenesis. TEAD2 is significantly overexpressed in tumor samples with lower overall survival rate in TCGA Liver Hepatocellular Carcinoma database (Joo et al., 2020). Intriguingly, VGLL4, but not YAP/TAZ, is correlated with TEAD2 in tumor samples, suggesting TEAD2 modulates HCC progression in a YAP-independent manner. By contrast, two SNPs of TEAD3 (rs11756089 CT/TT, rs2076173 TC/CC) are associated with superior survival rate in cancer patients (Xia et al., 2017). However, the expression level of TEADs was not evaluated in this study and the possible mechanism involved requires further investigated.

#### Breast Cancer

Although targeting the estrogen receptor (ER) or epithelia growth factor receptor (EGFR) signaling is a foremost strategy for breast cancer therapy, emerging data shows that the Hippo pathway promotes the breast cancer progression in various aspects, including cancer cell proliferation, migration, evasion and most importantly therapy resistance (Lamar et al., 2012; Wu and Yang, 2018). Increased copy number and upregulated expression of TEAD4 were reported in triple negative breast cancers (TNBC) (Han et al., 2008). It has been shown that YAP/TEAD4 complex acts as ERα cofactor and regulates E_2_/ERα target genes to enhancer activation in a non-canonical Hippo pathway (Zhu et al., 2019). In addition, TEAD4 also binds to the enhancer region of TRAM2 (Translocation Associated Membrane Protein 2), a key regulator of cell proliferation and invasion (Li L. et al., 2021). TEAD4 interacts with KLF5 (Kruppel Like Factor 5) to suppress CDK inhibitor p27 expression, resulting in the cancer cell growth (Wang C. et al., 2015). KLF5 also induces the expression of a lncRNA IGFL2-AS1 (IGFL2 Antisense RNA 1) to form a transcriptional complex with TEAD4 and promotes the IGFL1 (Insulin growth factor-like family member 1) gene expression (Wang H. et al., 2021). He et al. showed that glucocorticoids *via* glucocorticoid receptor (GR) transcriptionally activates TEAD4 independent of YAP/TAZ. TEAD4 expression correlates not only positively with GR expression in breast cancer, but also with poor survival and metastasis (He et al., 2019). These studies suggest the contribution of TEAD4 to the breast cancer progression, especially therapy resistance, hence targeting TEAD4 should be a new avenue to improve the breast cancer therapy.

#### Colon Cancer

Under normal circumstance, YAP plays a major role in digestive system, especially in tissue regeneration for maintaining the intestinal homeostasis (Yu et al., 2015). Cai et al. showed that YAP is upregulated in regenerating crypts. Vice versa, the regeneration is impaired in knockout YAP crypts, suggesting the oncogenic role of YAP in colon cancer (Cai et al., 2010). In colorectal cancer, YAP/TEAD4 cooperates with AP-1 and the p160 family of steroid receptor coactivator SRC1-3 to regulate the genes associated with tumor migration and invasion (Liu X. et al., 2016). Interestingly, TEAD4 is able to transcriptionally induce YAP expression to form a feedforward loop and promote tumorigenesis (Tang et al., 2018). YAP/TEAD4 induces the RAN binding protein 1 (RANBP1), which is a critical regulator of pre-miRNA nuclear exporter, exportin-5. Overexpression of exportin-5 increases the level of microRNAs that target LATS1/2, resulting in a negative feedback on Hippo signal pathway (Zheng et al., 2022). Nevertheless, TEAD4 also can modulate the colon cancer progression in a YAP-independent manner. In colorectal cancer, TEAD4 targets EMT genes and promotes metastasis. In this case, YAP was apparently not involved as TEAD4 Y429H mutant which fails to bind YAP can stimulate vimentin expression and promote metastasis like the wild type (Liu Y. et al., 2016). TEAD4’s nuclear expression can be used as a biomarker for colorectal cancer progression and poor prognosis (Liu Y. et al., 2016). Similarly, the overexpression of TEAD4 direct target gene SIX1 (sine oculis 1) correlates with poor prognosis of colorectal cancer patients (Yu et al., 2021). The underlying mechanism of both YAP-dependent and independent nodules on TEAD4 regulation should further elucidated to improve the cancer treatment.

#### Gastric Cancer

In gastric cancer, TEAD4 promotes cell cycle G1/S progression by increasing the expression of cyclins (D1 and E1) and CDKs (CDK2, 4, 6) (Teng et al., 2016). It also augments the transcription of a lncRNA MNX1-AS1 which sequesters BCL2-targeting miR-6785-50, leading to up-regulation of pro-survival BCL2 (B-cell lymphoma 2) protein (Shuai et al., 2020). An additional direct target of YAP/TEAD4 is a nucleotide sugar transporter, SLC35B4, which plays a key role in cancer metabolism and cancer cell proliferation (Liu J. et al., 2019). Hypo-methylation at CpG sites of TEAD4 promoter causing TEAD4 overexpression in gastric cancer tissues resulted in larger tumor size and lower survival rates (Lim et al., 2014). The reduced expression of TEAD4-targeting miR-4269 leads to increased nuclear TEAD4 and contributes to poor prognosis (Zhou et al., 2017). It should be noted that, both TEAD1 and TEAD4 are overexpressed in gastric cancer TCGA cohort in this study (Zhou et al., 2017). However, overexpressed TEAD1 is only observed in two subtypes of gastric cancer, Epstein–Barr virus-positive subtype and microsatellite instability subtype. By contrast, TEAD4 overexpression is observed in all four gastric cancer types, including additional gnomically stable subtype and chromosomal instability subtype, suggesting TEAD4 may have higher impact on gastric cancer progression.

#### Brain Tumor

The Hippo pathway plays a key role and can be regulated by multiple signaling pathways in brain tumor (Masliantsev et al., 2021). In gliomas, the ortholog TAZ is a dominant form and frequently overexpressed and associated with its aggressiveness (Zhang et al., 2016). In high grade gliomas, TAZ binds TEAD4 and modulates the expression of cyclin D1, Bcl-2 and MMP-9 (Matrix metallopeptidase 9), leading to cell proliferation and migration (Li et al., 2016). Additionally, high TEAD4 DNA copy number variation with isocitrate dehydrogenase mutations in low grade glioma correlates with a shorter overall survival and disease-free survival (Yuan et al., 2021). In addition to TEAD4, TEAD2 also can interact with YAP/TAZ and modulate the malignancy in brain tumor (Bhat et al., 2011; Lu et al., 2017). Indeed, disrupting the interaction of YAP with TEADs suppresses the tumor progression (Saunders et al., 2021).

#### Ovarian Cancer

Elevated YAP expression has been reported in different subtypes of ovarian cancer and associated with poor prognosis (Xia et al., 2014a). It has been reported that copy number increase and overexpression of TEAD4 are detected in ovarian cancer and fallopian tube carcinoma (Nowee et al., 2007). More importantly, among four TEAD members, TEAD4 is co-expressed with YAP in all subtypes of ovarian cancer. Silencing of YAP in ovarian cancer cell lines increases the drug sensitivity in both *in vitro* and *in vivo* xenograft model (Xia et al., 2014a). By contrast, overexpression of YAP/TEAD4 contribute to chemo-drug resistance, migration, and growth of ovarian cancer (Xia et al., 2014b). Ren et al. showed that TEAD4 expression not only has a higher diagnostic value with better sensitivity and lower false positive rate than TEAD1-3 in ovarian serous carcinoma patients, but also negatively correlates with the tumor-infiltrating immune cells (Ren et al., 2021). These results suggest that disrupting the interaction of YAP/TEAD4 or direct targeting TEAD4 could be a potential strategy for ovarian cancer therapy.

#### Bladder Cancer

Increasing evidence shows that the Hippo pathway plays a key role in bladder cancer progression (Xia et al., 2018). YAP is overexpressed and associated with poor prognosis of bladder cancer (Liu et al., 2013). Inhibition of YAP activity suppresses bladder cancer growth and invasion (Dong et al., 2018). The multivariate Cox regression analysis showed that TEAD4 is an independent prognostic factor for bladder cancer. Intriguing, infiltrating immune cells including CD4^+^ T cells, NK (natural killer) cells, macrophages, and neutrophils were positively correlated with the expression of TEAD4 that implicates immune cells in bladder tumor microenvironment play a promoting role in tumor progression (Wang J. et al., 2021). Furthermore, knockdown of TEAD4 Inhibits bladder cancer cells migration and invasion (Huang et al., 2021).

#### Prostate Cancer

The Hippo pathway plays a key role in prostate cancer progression at different stages (Salem and Hansen, 2019). At the early stage, ETS-regulated gene (ERG) induces YAP expression, which drives the YAP/TEAD4 transcriptional complex to induce the development of prostate cancer. Moreover, Wnt signaling induces the interaction of YAP with AR (androgen receptor) to drive the expression of downstream targets (Seo et al., 2017). Whether TEAD members were involved in AR mediated oncogenesis is not clear in this study. Recent work further showed that TEAD4 expression correlated with prostate cancer progression (Chen et al., 2021). The authors also demonstrated that the overexpressed TEAD4 regulates OXPHOS gene expression and modulates the mitochondrial functions in a YAP-independent manner. In addition to TEAD4, TEAD1 expression is also associated with prostate cancer progression (Knight et al., 2008). Silencing of TEAD1 suppresses the PCa cell proliferation *in vitro*.

#### Head and Neck Cancer

In the genomic profiling of head and neck cancer patients, the Hippo pathway is one of common pathways with most frequent genomic mutation and highly associated with metastasis and recurrence (Nisa et al., 2018). The overexpression of YAP is observed in HNSCC (head and neck squamous cell carcinomas) patients (Ge et al., 2011). TEAD4 expression is associated with HNSCC poor progression, such as pathological grade, clinical stage and metastasis (Zhang et al., 2018a). This study further showed that TEAD4 expression is induced by TGFβ1 to promote EMT in HNSCC.

#### Endometrial Cancer

TAZ is dominantly overexpressed in different subtypes of endometrial cancer to regulate the malignancy of endometrial cancer (Romero-Perez et al., 2015). Several studies show that the Hippo pathway modulate the endometrial cancer progression via cross talk with other signaling pathways (Wang et al., 2016a; Wang et al., 2017; Wen et al., 2020). Inactivation of YAP activity by verteporfin treatment suppressed cell proliferation and invasion and induced apoptosis in endometrial cancer cells (Dasari et al., 2017). Hu et al. showed that ARID1A (AT-rich interactive domain-containing protein 1A), a key subunit of SWI/SNF complex, recruits FOXA1 (Forkhead Box A1) and TEAD4 to regulate ER binding on circadian rhythm genes in ER-positive endometrial cancer (Hu et al., 2020). The transcriptional complex of TEAD4 and AP-1 controls cell migration and invasion by regulating its downstream targets such as CDH2 (Cadherin 2) and MACF1 (Microtubule Actin Crosslinking Factor 1) in endometrial cancer and other cancers (Liu X. et al., 2016).

#### Esophageal Cancer

The role of Hippo pathway in esophageal cancer (EC) progression has been recognized recently. Xu et al. demonstrated YAP is overexpressed in different EC datasets, including those of cancer patients and EC cancer cell lines. The transcriptional YAP/TEAD complex induces IRS2 (Insulin Receptor Substrate 2) expression through JNK/c-Jun pathway (Xu et al., 2021) However, which TEAD member is involved in this machinery was not identified in this study. Silencing of serum/glucocorticoid regulated kinase 1 (SGK1) disrupts the transcriptional YAP/TEAD4 complex, leading to the suppression of cancer cell growth and migration *in vivo* and *in vitro* (He et al., 2021). Another study showed that KIF4A (Kinesin Family Member 4A), a prognostic marker for EC, is driven by YAP/TEAD4 complex, which contributes to the EMT and anti-apoptotic property (Li Y. et al., 2021).

#### Renal Cell Carcinoma

YAP is reported to be overexpressed in clear cell renal cell carcinoma (ccRCC), the major type of renal cell carcinoma, and associated with cancer progression and poor prognosis (Rybarczyk et al., 2017). Schütte et al. found that elevated YAP expression is co-expressed with TEAD1 in ccRCC cell lines (Schutte et al., 2014). In this study, YAP and TEAD1 are co-localized at the promoter regions of CTGF (CCCTC-Binding Factor), MYC, EDN1 (Endothelin 1), and EDN2, leading to cancer cell proliferation and migration *in vitro* as well as tumor growth in xenograft model. Another study showed that tumor suppressor SAV1 is downregulated, which results in the activation the YAP1-TEAD3 complex in high-grade renal cell carcinoma (Matsuura et al., 2011). Of note is a recent study of ccRCC TCGA datasets analysis, which reveals that TEAD4, compared to other three TEAD members, shows the most significant correlation with ccRCC malignancy and cancer progression (Li et al., 2022). Silencing of TEAD4 suppresses cell proliferation in ccRCC cells *in vitro* and tumor growth *in vivo*.

#### Thyroid Cancer

In thyroid cancer, YAP expression is associated with poor prognosis and YAP controls cancer cell proliferation (Liu et al., 2017; Liu Z. et al., 2019). It was shown that the Ras expression is transcriptionally regulated by YAP/TEAD1 complex, which leads to thyroid tumorigenesis (Garcia-Rendueles et al., 2015). By contrast, Zhang et al. showed that TEAD4 seems to play a tumor suppressor role in thyroid cancer. Overexpressed TEAD4 suppresses thyroid cancer progression and metastasis *via* maintaining an appropriate Wnt signaling by upregulating Wnt3a (Zhang et al., 2022). The role of each TEAD member and its interaction with YAP or other co-factors would be necessary to clarify the role of TEADs in thyroid cancer.

### TEAD4 as a Potential Therapeutic Target

As a DNA anchoring protein without enzyme activity, TEAD4 is considered “non-druggable” in the traditional sense. Several strategies have been developed to get around this issue (Table 2). Given the overwhelming role of YAP in activating TEAD4’s transcriptional prowess, agents which disrupts YAP/TEAD4 interactions have been developed. One example is cyclic YAP-like peptides (Peptide 17 or Peptide 10), based on YAP-binding site to TEADs (YAP^81-100^ PQTVPMRLRKLPDSFFKPPE) (Zhang et al., 2014; Zhou et al., 2015). As VGLL4 is also a major coactivator of TEAD4, super-TDU was designed to mimic TEAD4 binding domains (TDU domain) of VGLL4, which competes the binding activity of YAP/TEAD4 (Jiao et al., 2014). The second strategy is target TEAD’s palmitoylation site, as this post-translational modification is required for TEAD activities. Pobbati et al. showed that NSAIDs (Non-steroidal anti-inflammatory drugs), such as flufenamic acid and niflumic acid inhibitors that bind to this site to decrease TEAD transcriptional activity without disrupting TEAD-YAP interaction (Pobbati et al., 2015). Bromofenamic acid also targets the TEAD palmitate-binding pocket and interrupts the interaction of TEAD2 and YAP (Pobbati et al., 2015). TED-347 and K-975 covalently bind to a cysteine residue in the palmitate-binding pocket of TEAD, which also inhibits protein-protein interaction between TEAD and YAP (Bum-Erdene et al., 2019; Kaneda et al., 2020). VT3989 (Vivace Therapeutics, Inc.) targets TEAD allosteric site, resulting in active site conformational change which inhibits palmitoylation and thus disrupts YAP/TEAD transcriptional activity. It is currently under clinical trial for metastatic solid tumors enriched for tumors with NF2 gene mutations (NCT04665206) (Barry et al., 2021). A third strategy targets YAP for degradation or reduced activity and expression. Verteporfin, a photosensitizing agent derived from porphyrin, was initially a regime for macular degeneration, then identified as an inhibitor for YAP/TEAD4 interaction (Liu-Chittenden et al., 2012). Wang et al. later showed that verteporfin suppresses YAP function through the induction of 14-3-3σ, resulting in YAP degradation (Wang et al., 2016b). Verteporfin has been shown the anti-tumor effect in various cancers (Wei and Li, 2020) and currently under the clinical trial for recurrent glioblastoma (NCT04590664) (Vigneswaran et al., 2021). Similarly, Pazopanib, MF-438 and XAV939 inhibit YAP function by induction of YAP degradation (Wang W. et al., 2015; Noto et al., 2017). Dasatinib, Pazopanib and Cerivastatin induce YAP phosphorylation to suppress YAP activity (Oku et al., 2015). CA3 downregulates YAP protein expression and its transcriptional activity (Song et al., 2018). C19 induces phosphorylation and activation of MST1 (Macrophage Stimulating 1) and LATS1/2 of Hippo pathway, which results in the degradation of the YAP homolog, TAZ (Basu et al., 2014). A combination of epigenetic regulators’ inhibitors, BET (bromodomain and extraterminal protein) and HDAC (histone deacetylase), suppresses YAP and AKT expression and induces apoptosis in melanoma cells (Heinemann et al., 2015).

**TABLE 2:** Summary of TEADs inhibitors.

Drug (brand Name)	Functions	References
Cyclic YAP-like peptides (Peptide 17 or 10)	Targets YAP’s binding site to TEADs	Zhang et al. (2014), Zhou et al. (2015)
Super-TDU	Mimicking TEAD4 binding domain (TDU) of VGLL4 and interrupts YAP-TEAD4 interaction	Jiao et al. (2014)
Flufenamic acid (TED-346), Niflumic acid	TEAD palmitate-binding pocket NO effect on YAP/TEAD interaction	Pobbati et al. (2015)
Bromofenamic acid	TEAD palmitate-binding pocket YAP-TEAD2 interaction	Pobbati et al. (2015)
TED-347	TEAD palmitate-binding pocket YAP-TEAD interaction	Bum-Erdene et al., 2019
K-975	TEAD palmitate-binding pocket YAP-TEAD interaction	Kaneda et al. (2020)
VT3989	TEAD palmitate-binding pocket YAP-TEAD interaction	Barry et al. (2021)
Verteporfin (Visudyne, Norvatis)	Up-regulation of 14-3-3σ sequestering YAP	Liu-Chittenden et al., 2012, Wang et al., 2016b, Vigneswaran et al. (2021)
Dasatinib, Pazopanib	Both induce YAP phosphorylation. Pazopanib induces YAP degradation	Oku et al. (2015)
MF-438	YAP/TAZ degradation	Noto et al. (2017)
XAV939	YAP degradation	Wang W. et al., 2015, Kurppa et al. (2020)
Cerivastatin	YAP phosphorylation	Sorrentino et al. (2014)
CA3	Inhibits YAP expression	Song et al. (2018)
C19	Activates MST1 and LATS1/2	Basu et al. (2014)
BET151 (BET inhibitor) +Panobinostat (HDAC inhibitor)	Downmodulates AKT and YAP signaling pathways	Heinemann et al. (2015)

## Conclusion and Perspectives

Based on the accumulated evidence described above, it is clear that TEAD4 is a pivotal transcription factor in development and disease. In this review, we have emphasized the unique roles of TEAD4 and as such, TEAD4’s shared functions with other TEADs are not described. TEAD4 thus has even larger role in these processes. As cited above, there are several excellent reviews on this topic. TEAD4’s ability to transcribe both mitochondrial and nuclear-encoded OXPHOS genes is unique, which plays a key role in maintaining mitochondrial OXPHOS integrity to avoid excessive ROS production, in the face of strong “Warburg effect” in cancer cells. This property is specific to cancer cells and targeting TEAD4 would preferentially kill cancer cells. The current therapies targeting TEAD4 action largely focus on the disruption of YAP/TEAD4 interaction or YAP pathway, which is understandable, as YAP is the major coactivator of TEAD4. Yet, there are also YAP-independent action of TEAD4, including the activation of OXPHOS gene, which would not be affected. Strategy to degrade TEAD4 itself or diminish its expression could be considered. As TEAD4 knockout mice are viable after embryo implantation stage and with functional redundancy with other TEAD members (Yagi et al., 2007), TEAD4 knockout may specifically affect cancer cells. In preclinical studies, targeting TEAD4 by CRISPR or shRNA provides proof of concept evidence that this may work (Chen et al., 2021). In clinics, an emerging modality is the application of ASO (antisense oligos) techniques, encouraged by recent success in the treatment of spinal muscular atrophy (Corey, 2017) and the efficient delivery of mRNA-based COVID vaccine into cells (Corbett et al., 2020; Polack et al., 2020). This strategy deserves more attention. Another outstanding question is how TEAD4, but not other TEAD family members, is translocated into mitochondria, and how it uniquely affects mitochondrial functions and biogenesis. *In vitro* studies showed that the mitochondrial morphology of TEAD4 knock-out cancer cells is severely damaged and mitochondrial functions suppressed. There is a growing interest in targeting mitochondrial pathways for cancer therapy (DeBerardinis and Chandel, 2020). Targeting TEAD4’s translocation ability can also be considered. Finally, the strong link between TEAD4 and super-enhancers are provocative. Knowledge about the molecular details of this process will increase our fundamental understanding of super enhancers or oncogenic super enhancers.

## References

[B1] Barry E. R. Simov V. Valtingojer I. Venier O. (2021). Recent Therapeutic Approaches to Modulate the Hippo Pathway in Oncology and Regenerative Medicine. Cells 10, 2715. 10.3390/cells10102715 34685695 PMC8534579

[B2] Basu D. Lettan R. Damodaran K. Strellec S. Reyes-Mugica M. Rebbaa A. (2014). Identification, Mechanism of Action, and Antitumor Activity of a Small Molecule Inhibitor of Hippo, TGF-β, and Wnt Signaling Pathways. Mol. Cancer Ther. 13, 1457–1467. 10.1158/1535-7163.mct-13-0918 24694946

[B3] Bhat K. P. L. Salazar K. L. Balasubramaniyan V. Wani K. Heathcock L. Hollingsworth F. (2011). The Transcriptional Coactivator TAZ Regulates Mesenchymal Differentiation in Malignant Glioma. Genes Dev. 25, 2594–2609. 10.1101/gad.176800.111 22190458 PMC3248681

[B4] Bum-Erdene K. Zhou D. Gonzalez-Gutierrez G. Ghozayel M. K. Si Y. Xu D. (2019). Small-Molecule Covalent Modification of Conserved Cysteine Leads to Allosteric Inhibition of the TEAD⋅Yap Protein-Protein Interaction. Cel Chem. Biol. 26, 378–389. 10.1016/j.chembiol.2018.11.010 30581134

[B5] Cai J. Zhang N. Zheng Y. de Wilde R. F. Maitra A. Pan D. (2010). The Hippo Signaling Pathway Restricts the Oncogenic Potential of an Intestinal Regeneration Program. Genes Dev. 24, 2383–2388. 10.1101/gad.1978810 21041407 PMC2964748

[B6] Cai W.-Y. Lin L.-Y. Hao H. Zhang S.-M. Ma F. Hong X.-X. (2017). Yes-associated Protein/TEA Domain Family Member and Hepatocyte Nuclear Factor 4-alpha (HNF4α) Repress Reciprocally to Regulate Hepatocarcinogenesis in Rats and Mice. Hepatology 65, 1206–1221. 10.1002/hep.28911 27809333

[B7] Cao X. Pfaff S. L. Gage F. H. (2008). YAP Regulates Neural Progenitor Cell Number via the TEA Domain Transcription Factor. Genes Dev. 22, 3320–3334. 10.1101/gad.1726608 19015275 PMC2600760

[B8] Chan P. Han X. Zheng B. Deran M. Yu J. Jarugumilli G. K. (2016). Autopalmitoylation of TEAD Proteins Regulates Transcriptional Output of the Hippo Pathway. Nat. Chem. Biol. 12, 282–289. 10.1038/nchembio.2036 26900866 PMC4798901

[B9] Chen C.-L. Hsu S.-C. Chung T.-Y. Chu C.-Y. Wang H.-J. Hsiao P.-W. (2021). Arginine Is an Epigenetic Regulator Targeting TEAD4 to Modulate OXPHOS in Prostate Cancer Cells. Nat. Commun. 12, 2398. 10.1038/s41467-021-22652-9 33893278 PMC8065123

[B10] Chen L. Chan S. W. Zhang X. Walsh M. Lim C. J. Hong W. (2010). Structural Basis of YAP Recognition by TEAD4 in the Hippo Pathway. Genes Dev. 24, 290–300. 10.1101/gad.1865310 20123908 PMC2811830

[B11] Chen M. Huang B. Zhu L. Chen K. Liu M. Zhong C. (2020). Structural and Functional Overview of TEAD4 in Cancer Biology. Ott Vol. 13, 9865–9874. 10.2147/ott.s266649 PMC7547805 33116572

[B12] Cockburn K. Biechele S. Garner J. Rossant J. (2013). The Hippo Pathway Member Nf2 Is Required for Inner Cell Mass Specification. Curr. Biol. 23, 1195–1201. 10.1016/j.cub.2013.05.044 23791728

[B13] Corbett K. S. Flynn B. Foulds K. E. Francica J. R. Boyoglu-Barnum S. Werner A. P. (2020). Evaluation of the mRNA-1273 Vaccine against SARS-CoV-2 in Nonhuman Primates. N. Engl. J. Med. 383, 1544–1555. 10.1056/nejmoa2024671 32722908 PMC7449230

[B14] Corey D. R. (2017). Nusinersen, an Antisense Oligonucleotide Drug for Spinal Muscular Atrophy. Nat. Neurosci. 20, 497–499. 10.1038/nn.4508 28192393

[B15] Coto-Llerena M. Tosti N. Taha-Mehlitz S. Kancherla V. Paradiso V. Gallon J. (2021). Transcriptional Enhancer Factor Domain Family Member 4 Exerts an Oncogenic Role in Hepatocellular Carcinoma by Hippo-independent Regulation of Heat Shock Protein 70 Family Members. Hepatol. Commun. 5, 661–674. 10.1002/hep4.1656 33860124 PMC8034568

[B16] Dasari V. R. Mazack V. Feng W. Nash J. Carey D. J. Gogoi R. (2017). Verteporfin Exhibits YAP-independent Anti-proliferative and Cytotoxic Effects in Endometrial Cancer Cells. Oncotarget 8, 28628–28640. 10.18632/oncotarget.15614 28404908 PMC5438678

[B17] DeBerardinis R. J. Chandel N. S. (2020). We Need to Talk about the Warburg Effect. Nat. Metab. 2, 127–129. 10.1038/s42255-020-0172-2 32694689

[B18] Dong L. Lin F. Wu W. Liu Y. Huang W. (2018). Verteporfin Inhibits YAP-Induced Bladder Cancer Cell Growth and Invasion via Hippo Signaling Pathway. Int. J. Med. Sci. 15, 645–652. 10.7150/ijms.23460 29725256 PMC5930467

[B19] Edwards D. N. Ngwa V. M. Wang S. Shiuan E. Brantley-Sieders D. M. Kim L. C. (2017). The Receptor Tyrosine Kinase EphA2 Promotes Glutamine Metabolism in Tumors by Activating the Transcriptional Coactivators YAP and TAZ. Sci. Signal. 10, eaan4667. 10.1126/scisignal.aan4667 29208682 PMC5819349

[B20] Feng X. Wang Z. Wang F. Lu T. Xu J. Ma X. (2019). Dual Function of VGLL4 in Muscle Regeneration. EMBO J. 38, e101051. 10.15252/embj.2018101051 31328806 PMC6717915

[B21] Feng X. Lu T. Li J. Yang R. Hu L. Ye Y. (2020). The Tumor Suppressor Interferon Regulatory Factor 2 Binding Protein 2 Regulates Hippo Pathway in Liver Cancer by a Feedback Loop in Mice. Hepatology 71, 1988–2004. 10.1002/hep.30961 31538665

[B22] Figeac N. Mohamed A. D. Sun C. Schönfelder M. Matallanas D. Garcia-Munoz A. (2019). VGLL3 Operates via TEAD1, TEAD3 and TEAD4 to Influence Myogenesis in Skeletal Muscle. J. Cel Sci 132, jcs225946. 10.1242/jcs.225946 PMC6633393 31138678

[B23] Frum T. Watts J. L. Ralston A. (2019). TEAD4, YAP1 and WWTR1 Prevent the Premature Onset of Pluripotency Prior to the 16-cell Stage. Development 146, dev179861. 10.1242/dev.179861 31444221 PMC6765126

[B24] Fujii M. Toyoda T. Nakanishi H. Yatabe Y. Sato A. Matsudaira Y. (2012). TGF-β Synergizes with Defects in the Hippo Pathway to Stimulate Human Malignant Mesothelioma Growth. J. Exp. Med. 209, 479–494. 10.1084/jem.20111653 22329991 PMC3302232

[B25] Gao J. Zhang Y. Chen H. Chen Q. Feng D. Zhang L. (2019). Computational Insights into the Interaction Mechanism of Transcription Cofactor Vestigial-like Protein 4 Binding to TEA Domain Transcription Factor 4 by Molecular Dynamics Simulation and Molecular Mechanics Generalized Born/surface Area) Calculation. J. Biomol. Struct. Dyn. 37, 2538–2545. 10.1080/07391102.2018.1491889 30051771

[B26] Garcia-Rendueles M. E. R. Ricarte-Filho J. C. Untch B. R. Landa I. Knauf J. A. Voza F. (2015). NF2 Loss Promotes Oncogenic RAS-Induced Thyroid Cancers via YAP-dependent Transactivation of RAS Proteins and Sensitizes Them to MEK Inhibition. Cancer Discov. 5, 1178–1193. 10.1158/2159-8290.cd-15-0330 26359368 PMC4642441

[B27] Ge L. Smail M. Meng W. Shyr Y. Ye F. Fan K.-H. (2011). Yes-associated Protein Expression in Head and Neck Squamous Cell Carcinoma Nodal Metastasis. PLoS One 6, e27529. 10.1371/journal.pone.0027529 22096589 PMC3212574

[B28] Haider S. Meinhardt G. Saleh L. Fiala C. Pollheimer J. Knöfler M. (2016). Notch1 Controls Development of the Extravillous Trophoblast Lineage in the Human Placenta. Proc. Natl. Acad. Sci. U.S.A. 113, E7710–e7719. 10.1073/pnas.1612335113 27849611 PMC5137701

[B29] Han W. Jung E.-M. Cho J. Lee J. W. Hwang K.-T. Yang S.-J. (2008). DNA Copy Number Alterations and Expression of Relevant Genes in Triple-Negative Breast Cancer. Genes Chromosom. Cancer 47, 490–499. 10.1002/gcc.20550 18314908

[B30] Hau J. C. Erdmann D. Mesrouze Y. Furet P. Fontana P. Zimmermann C. (2013). The TEAD4-YAP/TAZ Protein-Protein Interaction: Expected Similarities and Unexpected Differences. Chembiochem 14, 1218–1225. 10.1002/cbic.201300163 23780915

[B150] Hashimoto S. Furukawa S. Hashimoto A. Tsutaho A. Fukao A. Sakamura Y. (2019). ARF6 and AMAP1 are Major Targets of KRAS and TP53 Mutations to Promote Invasion, PD-L1 Dynamics, and Immune Evasion of Pancreatic Cancer. Proc. Natl. Acad. Sci. U S A 116 (35), 17450–17459. 31399545 10.1073/pnas.1901765116 PMC6717289

[B31] Hazan I. Monin J. Bouwman B. A. M. Crosetto N. Aqeilan R. I. (2019). Activation of Oncogenic Super-enhancers Is Coupled with DNA Repair by RAD51. Cel Rep. 29, 560–572. e4. 10.1016/j.celrep.2019.09.001 PMC6899447 31618627

[B32] He L. Yuan L. Sun Y. Wang P. Zhang H. Feng X. (2019). Glucocorticoid Receptor Signaling Activates TEAD4 to Promote Breast Cancer Progression. Cancer Res. 79, 4399–4411. 10.1158/0008-5472.can-19-0012 31289134

[B33] He S. Zhang H. Xiao Z. Bhushan S. Gao K. Wang W. (2021). The Interaction of TEA Domain Transcription Factor 4 (TEAD4) and Yes-Associated Protein 1 (YAP1) Promoted the Malignant Process Mediated by Serum/glucocorticoid Regulated Kinase 1 (SGK1). Bioengineered 12, 601–614. 10.1080/21655979.2021.1882142 33517828 PMC8806348

[B34] Heinemann A. Cullinane C. de Paoli-Iseppi R. Wilmott J. S. Gunatilake D. Madore J. (2015). Combining BET and HDAC Inhibitors Synergistically Induces Apoptosis of Melanoma and Suppresses AKT and YAP Signaling. Oncotarget 6, 21507–21521. 10.18632/oncotarget.4242 26087189 PMC4673282

[B35] Hirate Y. Cockburn K. Rossant J. Sasaki H. (2012). Tead4 Is Constitutively Nuclear, while Nuclear vs. Cytoplasmic Yap Distribution Is Regulated in Preimplantation Mouse Embryos. Proc. Natl. Acad. Sci. U S A. 109, 33899. author reply E3391-2. 10.1073/pnas.1211810109 PMC3528498 23169672

[B36] Home P. Saha B. Ray S. Dutta D. Gunewardena S. Yoo B. (2012). Altered Subcellular Localization of Transcription Factor TEAD4 Regulates First Mammalian Cell Lineage Commitment. Proc. Natl. Acad. Sci. U.S.A. 109, 7362–7367. 10.1073/pnas.1201595109 22529382 PMC3358889

[B37] Hu H. Chen Z. Ji L. Wang Y. Yang M. Lai R. (2020). ARID1A-dependent Permissive Chromatin Accessibility Licenses Estrogen-Receptor Signaling to Regulate Circadian Rhythms Genes in Endometrial Cancer. Cancer Lett. 492, 162–173. 10.1016/j.canlet.2020.08.034 32858102

[B151] Hu Y. Mu H. Deng Z. (2021). The Transcription Factor TEAD4 Enhances Lung Adenocarcinoma Progression Through Enhancing PKM2 Mediated Glycolysis. Cell Biol. Int. 45 (10), 2063–2073. 34196069 10.1002/cbin.11654

[B38] Huang Z. Yan Y. Tang P. Cai J. Cao X. Wang Z. (2021). TEAD4 as a Prognostic Marker Promotes Cell Migration and Invasion of Urinary Bladder Cancer via EMT. Ott Vol. 14, 937–949. 10.2147/ott.s290425 PMC7882801 33603398

[B39] Huh H. D. Kim D. H. Jeong H. S. Park H. W. (2019). Regulation of TEAD Transcription Factors in Cancer Biology. Cells 8, 600. 10.3390/cells8060600 PMC6628201 31212916

[B40] Hwang J. J. Chambon P. Davidson I. (1993). Characterization of the Transcription Activation Function and the DNA Binding Domain of Transcriptional Enhancer Factor-1. EMBO J. 12, 2337–2348. 10.1002/j.1460-2075.1993.tb05888.x 8389695 PMC413464

[B41] ICGC/TCGA Pan-Cancer Analysis of Whole Genomes Consortium (2020). Pan-cancer Analysis of Whole Genomes. Nature 578, 82–93. 10.1038/s41586-020-1969-6 32025007 PMC7025898

[B42] Jang J.-W. Kim M.-K. Lee Y.-S. Lee J.-W. Kim D.-M. Song S.-H. (2017). RAC-LATS1/2 Signaling Regulates YAP Activity by Switching between the YAP-Binding Partners TEAD4 and RUNX3. Oncogene 36, 999–1011. 10.1038/onc.2016.266 27425596

[B43] Jiang S.-W. Desai D. Khan S. Eberhardt N. L. (2000). Cooperative Binding of TEF-1 to Repeated GGAATG-Related Consensus Elements with Restricted Spatial Separation and Orientation. DNA Cel Biol. 19, 507–514. 10.1089/10445490050128430 10975468

[B44] Jiao S. Guan J. Chen M. Wang W. Li C. Wang Y. (2018). Targeting IRF3 as a YAP Agonist Therapy against Gastric Cancer. J. Exp. Med. 215, 699–718. 10.1084/jem.20171116 29339449 PMC5789414

[B45] Jiao S. Li C. Hao Q. Miao H. Zhang L. Li L. (2017). VGLL4 Targets a TCF4-TEAD4 Complex to Coregulate Wnt and Hippo Signalling in Colorectal Cancer. Nat. Commun. 8, 14058. 10.1038/ncomms14058 28051067 PMC5216127

[B46] Jiao S. Wang H. Shi Z. Dong A. Zhang W. Song X. (2014). A Peptide Mimicking VGLL4 Function Acts as a YAP Antagonist Therapy against Gastric Cancer. Cancer Cell 25, 166–180. 10.1016/j.ccr.2014.01.010 24525233

[B47] Joo J. S. Cho S. Y. Rou W. S. Kim J. S. Kang S. H. Lee E. S. (2020). TEAD2 as a Novel Prognostic Factor for Hepatocellular Carcinoma. Oncol. Rep. 43, 1785–1796. 10.3892/or.2020.7578 32323824 PMC7160555

[B48] Kaan H. Y. K. Chan S. W. Tan S. K. J. Guo F. Lim C. J. Hong W. (2017). Crystal Structure of TAZ-TEAD Complex Reveals a Distinct Interaction Mode from that of YAP-TEAD Complex. Sci. Rep. 7, 2035. 10.1038/s41598-017-02219-9 28515457 PMC5435683

[B49] Kaneda A. Seike T. Danjo T. Nakajima T. Otsubo N. Yamaguchi D. (2020). The Novel Potent TEAD Inhibitor, K-975, Inhibits YAP1/TAZ-TEAD Protein-Protein Interactions and Exerts an Anti-tumor Effect on Malignant Pleural Mesothelioma. Am. J. Cancer Res. 10, 4399–4415. 33415007 PMC7783735

[B50] Kaneko K. J. DePamphilis M. L. (2013). TEAD4 Establishes the Energy Homeostasis Essential for Blastocoel Formation. Development 140, 3680–3690. 10.1242/dev.093799 23903192 PMC3742148

[B51] Kim N.-G. Gumbiner B. M. (2019). Cell Contact and Nf2/Merlin-dependent Regulation of TEAD Palmitoylation and Activity. Proc. Natl. Acad. Sci. U.S.A. 116, 9877–9882. 10.1073/pnas.1819400116 31043565 PMC6525549

[B52] Knight J. F. Shepherd C. J. Rizzo S. Brewer D. Jhavar S. Dodson A. R. (2008). TEAD1 and C-Cbl Are Novel Prostate Basal Cell Markers that Correlate with Poor Clinical Outcome in Prostate Cancer. Br. J. Cancer 99, 1849–1858. 10.1038/sj.bjc.6604774 19002168 PMC2600693

[B53] Kumar R. P. Ray S. Home P. Saha B. Bhattacharya B. Wilkins H. M. (2018). Regulation of Energy Metabolism during Early Mammalian Development: TEAD4 Controls Mitochondrial Transcription. Development 145, dev162644. 10.1242/dev.162644 30201685 PMC6198476

[B152] Kurppa K. J. Liu Y. To C. Zhang T. Fan M. Vajdi A. (2020). Treatment-Induced Tumor Dormancy through YAP-Mediated Transcriptional Reprogramming of the Apoptotic Pathway. Cancer Cell. 37 (1), 104–122.e12. 31935369 10.1016/j.ccell.2019.12.006 PMC7146079

[B54] Lamar J. M. Stern P. Liu H. Schindler J. W. Jiang Z. G. Hynes R. O. (2012). The Hippo Pathway Target, YAP, Promotes Metastasis through its TEAD-Interaction Domain. Proc. Natl. Acad. Sci. U S A. 109, E2441–E2450. 10.1073/pnas.1212021109 22891335 PMC3443162

[B55] Li F. Feng Y. Jiang Q. Zhang J. Wu F. Li Q. (2022). Pan-cancer Analysis, Cell and Animal Experiments Revealing TEAD4 as a Tumor Promoter in ccRCC. Life Sci. 293, 120327. 10.1016/j.lfs.2022.120327 35065165

[B56] Li L. Ugalde A. P. Scheele C. L. G. J. Dieter S. M. Nagel R. Ma J. (2021). A Comprehensive Enhancer Screen Identifies TRAM2 as a Key and Novel Mediator of YAP Oncogenesis. Genome Biol. 22, 54. 10.1186/s13059-021-02272-8 33514403 PMC7845134

[B57] Li W. Dong S. Wei W. Wang G. Zhang A. Pu P. (2016). The Role of Transcriptional Coactivator TAZ in Gliomas. Oncotarget 7, 82686–82699. 10.18632/oncotarget.12625 27764783 PMC5347724

[B58] Li Y. Liu S. Ng E. Y. Li R. Poulsen A. Hill J. (2018). Structural and Ligand-Binding Analysis of the YAP-Binding Domain of Transcription Factor TEAD4. Biochem. J. 475, 2043–2055. 10.1042/bcj20180225 29760238

[B59] Li Y. Zhu X. Yang M. Wang Y. Li J. Fang J. (2021). YAP/TEAD4‐induced KIF4A Contributes to the Progression and Worse Prognosis of Esophageal Squamous Cell Carcinoma. Mol. Carcinogenesis 60, 440–454. 10.1002/mc.23303 34003522

[B60] Lim B. Park J.-L. Kim H.-J. Park Y.-K. Kim J.-H. Sohn H. A. (2014). Integrative Genomics Analysis Reveals the Multilevel Dysregulation and Oncogenic Characteristics of TEAD4 in Gastric Cancer. Carcinogenesis 35, 1020–1027. 10.1093/carcin/bgt409 24325916

[B61] Lin K. C. Moroishi T. Meng Z. Jeong H.-S. Plouffe S. W. Sekido Y. (2017a). Regulation of Hippo Pathway Transcription Factor TEAD by P38 MAPK-Induced Cytoplasmic Translocation. Nat. Cel Biol 19, 996–1002. 10.1038/ncb3581 PMC5541894 28752853

[B62] Lin K. C. Park H. W. Guan K.-L. (2017b). Regulation of the Hippo Pathway Transcription Factor TEAD. Trends Biochem. Sci. 42, 862–872. 10.1016/j.tibs.2017.09.003 28964625 PMC5735856

[B63] Liu J.-Y. Li Y.-H. Lin H.-X. Liao Y.-J. Mai S.-J. Liu Z.-W. (2013). Overexpression of YAP 1 Contributes to Progressive Features and Poor Prognosis of Human Urothelial Carcinoma of the Bladder. BMC Cancer 13, 349. 10.1186/1471-2407-13-349 23870412 PMC3750259

[B64] Liu J. Zhao X. Wang K. Zhang X. Yu Y. Lv Y. (2019). A Novel YAP1/SLC35B4 Regulatory axis Contributes to Proliferation and Progression of Gastric Carcinoma. Cell Death Dis 10, 452. 10.1038/s41419-019-1674-2 31175271 PMC6555804

[B65] Liu X. Li H. Rajurkar M. Li Q. Cotton J. L. Ou J. (2016). Tead and AP1 Coordinate Transcription and Motility. Cel Rep. 14, 1169–1180. 10.1016/j.celrep.2015.12.104 PMC4749442 26832411

[B66] Liu X. Zhao D. James L. Li J. Zeng H. (2011). Requirement of the Nuclear Localization of Transcription Enhancer Factor 3 for Proliferation, Migration, Tube Formation, and Angiogenesis Induced by Vascular Endothelial Growth Factor. FASEB j. 25, 1188–1197. 10.1096/fj.10-167619 21169383

[B67] Liu Y. Wang G. Yang Y. Mei Z. Liang Z. Cui A. (2016). Increased TEAD4 Expression and Nuclear Localization in Colorectal Cancer Promote Epithelial-Mesenchymal Transition and Metastasis in a YAP-independent Manner. Oncogene 35, 2789–2800. 10.1038/onc.2015.342 26387538

[B68] Liu Z. Zeng W. Wang S. Zhao X. Guo Y. Yu P. (2017). A Potential Role for the Hippo Pathway Protein, YAP, in Controlling Proliferation, Cell Cycle Progression, and Autophagy in BCPAP and KI Thyroid Papillary Carcinoma Cells. Am. J. Transl Res. 9, 3212–3223. 28804541 PMC5553873

[B69] Liu Z. Zeng W. Maimaiti Y. Ming J. Guo Y. Liu Y. (2019). High Expression of Yes-Activated Protein-1 in Papillary Thyroid Carcinoma Correlates with Poor Prognosis. Appl. Immunohistochem. Mol. Morphol. 27, 59–64. 10.1097/pai.0000000000000544 28682834

[B70] Liu-Chittenden Y. Huang B. Shim J. S. Chen Q. Lee S.-J. Anders R. A. (2012). Genetic and Pharmacological Disruption of the TEAD-YAP Complex Suppresses the Oncogenic Activity of YAP. Genes Dev. 26, 1300–1305. 10.1101/gad.192856.112 22677547 PMC3387657

[B71] Lu J. Yang Y. Guo G. Liu Y. Zhang Z. Dong S. (2017). IKBKE Regulates Cell Proliferation and Epithelial-Mesenchymal Transition of Human Malignant Glioma via the Hippo Pathway. Oncotarget 8, 49502–49514. 10.18632/oncotarget.17738 28548934 PMC5564784

[B72] Luo X. Zhang R. Lu M. Liu S. Baba H. A. Gerken G. (2021). Hippo Pathway Counter-regulates Innate Immunity in Hepatitis B Virus Infection. Front. Immunol. 12, 684424. 10.3389/fimmu.2021.684424 34113355 PMC8185339

[B73] Magico A. C. Bell J. B. (2011). Identification of a Classical Bipartite Nuclear Localization Signal in the Drosophila TEA/ATTS Protein Scalloped. PLoS One 6, e21431. 10.1371/journal.pone.0021431 21731746 PMC3121794

[B74] Mahoney W. M. JR. Hong J.-H. Yaffe M. B. Farrance I. K. G. (2005). The Transcriptional Co-activator TAZ Interacts Differentially with Transcriptional Enhancer Factor-1 (TEF-1) Family Members. Biochem. J. 388, 217–225. 10.1042/bj20041434 15628970 PMC1186710

[B75] Marquard S. Thomann S. Weiler S. M. E. Bissinger M. Lutz T. Sticht C. (2020). Yes-associated Protein (YAP) Induces a Secretome Phenotype and Transcriptionally Regulates Plasminogen Activator Inhibitor-1 (PAI-1) Expression in Hepatocarcinogenesis. Cell Commun Signal 18, 166. 10.1186/s12964-020-00634-6 33097058 PMC7583285

[B76] Masliantsev K. Karayan-Tapon L. Guichet P. O. (2021). Hippo Signaling Pathway in Gliomas. Cells 10, 184. 10.3390/cells10010184 33477668 PMC7831924

[B77] Matsuura K. Nakada C. Mashio M. Narimatsu T. Yoshimoto T. Tanigawa M. (2011). Downregulation of SAV1 Plays a Role in Pathogenesis of High-Grade clear Cell Renal Cell Carcinoma. BMC Cancer 11, 523. 10.1186/1471-2407-11-523 22185343 PMC3292516

[B78] Meinhardt G. Haider S. Kunihs V. Saleh L. Pollheimer J. Fiala C. (2020). Pivotal Role of the Transcriptional Co-activator YAP in Trophoblast Stemness of the Developing Human Placenta. Proc. Natl. Acad. Sci. U.S.A. 117, 13562–13570. 10.1073/pnas.2002630117 32482863 PMC7306800

[B79] Mesrouze Y. Bokhovchuk F. Meyerhofer M. Fontana P. Zimmermann C. Martin T. (2017). Dissection of the Interaction between the Intrinsically Disordered YAP Protein and the Transcription Factor TEAD. Elife 6, e25068. 10.7554/eLife.25068 28430104 PMC5400505

[B80] Mesrouze Y. Hau J. C. Erdmann D. Zimmermann C. Fontana P. Schmelzle T. (2014). The Surprising Features of the TEAD4-Vgll1 Protein-Protein Interaction. Chembiochem 15, 537–542. 10.1002/cbic.201300715 24504694

[B81] Moya I. M. Halder G. (2019). Hippo-YAP/TAZ Signalling in Organ Regeneration and Regenerative Medicine. Nat. Rev. Mol. Cel Biol 20, 211–226. 10.1038/s41580-018-0086-y 30546055

[B82] Nguyen-Lefebvre A. T. Selzner N. Wrana J. L. Bhat M. (2021). The Hippo Pathway: A Master Regulator of Liver Metabolism, Regeneration, and Disease. FASEB J. 35, e21570. 10.1096/fj.202002284RR 33831275

[B83] Nisa L. Barras D. Medová M. Aebersold D. M. Medo M. Poliaková M. (2018). Comprehensive Genomic Profiling of Patient-Matched Head and Neck Cancer Cells: A Preclinical Pipeline for Metastatic and Recurrent Disease. Mol. Cancer Res. 16, 1912–1926. 10.1158/1541-7786.mcr-18-0056 30108165

[B84] Nishioka N. Inoue K.-i. Adachi K. Kiyonari H. Ota M. Ralston A. (2009). The Hippo Signaling Pathway Components Lats and Yap Pattern Tead4 Activity to Distinguish Mouse Trophectoderm from Inner Cell Mass. Develop. Cel 16, 398–410. 10.1016/j.devcel.2009.02.003 19289085

[B85] Niu R.-J. Zheng Q.-C. Zhang H.-X. (2019). Molecular Dynamics Simulations Study of Influence of Tyr422Ala Mutation on Transcriptional Enhancer Activation Domain 4 (TEAD4) and Transcription Co-activators Complexes. J. Theor. Biol. 472, 27–35. 10.1016/j.jtbi.2019.04.009 30978352

[B86] Noland C. L. Gierke S. Schnier P. D. Murray J. Sandoval W. N. Sagolla M. (2016). Palmitoylation of TEAD Transcription Factors Is Required for Their Stability and Function in Hippo Pathway Signaling. Structure 24, 179–186. 10.1016/j.str.2015.11.005 26724994

[B87] Noto A. de Vitis C. Pisanu M. E. Roscilli G. Ricci G. Catizone A. (2017). Stearoyl-CoA-desaturase 1 Regulates Lung Cancer Stemness via Stabilization and Nuclear Localization of YAP/TAZ. Oncogene 36, 4573–4584. 10.1038/onc.2017.75 28368399

[B88] Nowee M. Snijders A. Rockx D. de Wit R. Kosma V. Hämäläinen K. (2007). DNA Profiling of Primary Serous Ovarian and Fallopian Tube Carcinomas with Array Comparative Genomic Hybridization and Multiplex Ligation-dependent Probe Amplification. J. Pathol. 213, 46–55. 10.1002/path.2217 17668415

[B89] Obier N. Cauchy P. Assi S. A. Gilmour J. Lie-A-Ling M. Lichtinger M. (2016). Cooperative Binding of AP-1 and TEAD4 Modulates the Balance between Vascular Smooth Muscle and Hemogenic Cell Fate. Development 143, 4324–4340. 10.1242/dev.139857 27802171 PMC5201045

[B90] Oku Y. Nishiya N. Shito T. Yamamoto R. Yamamoto Y. Oyama C. (2015). Small Molecules Inhibiting the Nuclear Localization of YAP/TAZ for Chemotherapeutics and Chemosensitizers against Breast Cancers. FEBS Open Bio 5, 542–549. 10.1016/j.fob.2015.06.007 PMC4506957 26199863

[B91] Park B. Chang S. Lee G.-J. Kang B. Kim J. K. Park H. (2019). Wnt3a Disrupts GR-TEAD4-Pparγ2 Positive Circuits and Cytoskeletal Rearrangement in a β-catenin-dependent Manner during Early Adipogenesis. Cel Death Dis 10, 16. 10.1038/s41419-018-1249-7 PMC6325140 30622240

[B92] Pobbati A. V. Han X. Hung A. W. Weiguang S. Huda N. Chen G.-Y. (2015). Targeting the Central Pocket in Human Transcription Factor TEAD as a Potential Cancer Therapeutic Strategy. Structure 23, 2076–2086. 10.1016/j.str.2015.09.009 26592798 PMC4660270

[B93] Polack F. P. Thomas S. J. Kitchin N. Absalon J. Gurtman A. Lockhart S. (2020). Safety and Efficacy of the BNT162b2 mRNA Covid-19 Vaccine. N. Engl. J. Med. 383, 2603–2615. 10.1056/nejmoa2034577 33301246 PMC7745181

[B94] Qiao Y. Wang Z. Tan F. Chen J. Lin J. Yang J. (2020). Enhancer Reprogramming within Pre-existing Topologically Associated Domains Promotes TGF-β-Induced EMT and Cancer Metastasis. Mol. Ther. 28, 2083–2095. 10.1016/j.ymthe.2020.05.026 32526202 PMC7474343

[B95] Quinn H. M. Vogel R. Popp O. Mertins P. Lan L. Messerschmidt C. (2021). YAP and β-Catenin Cooperate to Drive Oncogenesis in Basal Breast Cancer. Cancer Res. 81, 2116–2127. 10.1158/0008-5472.can-20-2801 33574090

[B96] Ren X. Wang X. Peng B. Liang Q. Cai Y. Gao K. (2021). Significance of TEAD Family in Diagnosis, Prognosis and Immune Response for Ovarian Serous Carcinoma. Ijgm Vol. 14, 7133–7143. 10.2147/ijgm.s336602 PMC8558638 34737608

[B97] Romero-Pérez L. Garcia-Sanz P. Mota A. Leskelä S. Hergueta-Redondo M. Díaz-Martín J. (2015). A Role for the Transducer of the Hippo Pathway, TAZ, in the Development of Aggressive Types of Endometrial Cancer. Mod. Pathol. 28, 1492–1503. 10.1038/modpathol.2015.102 26381823

[B98] Rybarczyk A. Klacz J. Wronska A. Matuszewski M. Kmiec Z. Wierzbicki P. M. (2017). Overexpression of the YAP1 Oncogene in clear Cell Renal Cell Carcinoma Is Associated with Poor Outcome. Oncol. Rep. 38, 427–439. 10.3892/or.2017.5642 28504812

[B99] Salem O. Hansen C. G. (2019). The Hippo Pathway in Prostate Cancer. Cells 8, 370. 10.3390/cells8040370 PMC6523349 31018586

[B100] Saunders J. T. Holmes B. Benavides-Serrato A. Kumar S. Nishimura R. N. Gera J. (2021). Targeting the YAP-TEAD Interaction Interface for Therapeutic Intervention in Glioblastoma. J. Neurooncol. 152, 217–231. 10.1007/s11060-021-03699-6 33511508 PMC8005474

[B101] Schütte U. Bisht S. Heukamp L. C. Kebschull M. Florin A. Haarmann J. (2014). Hippo Signaling Mediates Proliferation, Invasiveness, and Metastatic Potential of clear Cell Renal Cell Carcinoma. Translational Oncol. 7, 309–321. 10.1016/j.tranon.2014.02.005 PMC4101344 24913676

[B102] Seo W. I. Park S. Gwak J. Ju B. G. Chung J. I. Kang P. M. (2017). Wnt Signaling Promotes Androgen-independent Prostate Cancer Cell Proliferation through Up-Regulation of the Hippo Pathway Effector YAP. Biochem. Biophysical Res. Commun. 486, 1034–1039. 10.1016/j.bbrc.2017.03.158 28366633

[B103] Shi Z. He F. Chen M. Hua L. Wang W. Jiao S. (2017). DNA-binding Mechanism of the Hippo Pathway Transcription Factor TEAD4. Oncogene 36, 4362–4369. 10.1038/onc.2017.24 28368398

[B104] Shuai Y. Ma Z. Liu W. Yu T. Yan C. Jiang H. (2020). TEAD4 Modulated LncRNA MNX1-AS1 Contributes to Gastric Cancer Progression Partly through Suppressing BTG2 and Activating BCL2. Mol. Cancer 19, 6. 10.1186/s12943-019-1104-1 31924214 PMC6953272

[B105] Soncin F. Khater M. To C. Pizzo D. Farah O. Wakeland A. (2018). Comparative Analysis of Mouse and Human Placentae across Gestation Reveals Species-specific Regulators of Placental Development. Development 145, dev156273. 10.1242/dev.156273 29361559 PMC5825847

[B106] Song S. Xie M. Scott A. W. Jin J. Ma L. Dong X. (2018). A Novel YAP1 Inhibitor Targets CSC-Enriched Radiation-Resistant Cells and Exerts Strong Antitumor Activity in Esophageal Adenocarcinoma. Mol. Cancer Ther. 17, 443–454. 10.1158/1535-7163.mct-17-0560 29167315 PMC5805581

[B153] Sorrentino G. Ruggeri N. Specchia V. Cordenonsi M. Mano M. Dupont S. (2014). Metabolic Control of YAP and TAZ by the Mevalonate Pathway. Nat. Cell Biol. 16 (4), 357–366. 24658687 10.1038/ncb2936

[B107] Tang J.-Y. Yu C.-Y. Bao Y.-J. Chen L. Chen J. Yang S.-L. (2018). TEAD4 Promotes Colorectal Tumorigenesis via Transcriptionally Targeting YAP1. Cell Cycle 17, 102–109. 10.1080/15384101.2017.1403687 29157094 PMC5815434

[B108] Teng K. Deng C. Xu J. Men Q. Lei T. di D. (2016). Nuclear Localization of TEF3-1 Promotes Cell Cycle Progression and Angiogenesis in Cancer. Oncotarget 7, 13827–13841. 10.18632/oncotarget.7342 26885617 PMC4924681

[B109] Thomann S. Weiler S. M. E. Marquard S. Rose F. Ball C. R. Tóth M. (2020). YAP Orchestrates Heterotypic Endothelial Cell Communication via HGF/c-MET Signaling in Liver Tumorigenesis. Cancer Res. 80, 5502–5514. 10.1158/0008-5472.can-20-0242 33087321

[B110] Thomann S. Weiler S. M. E. Wei T. Sticht C. de la Torre C. Tóth M. (2021). YAP‐induced Ccl2 Expression Is Associated with a Switch in Hepatic Macrophage Identity and Vascular Remodelling in Liver Cancer. Liver Int. 41, 3011–3023. 10.1111/liv.15048 34459091

[B111] Totaro A. Panciera T. Piccolo S. (2018). YAP/TAZ Upstream Signals and Downstream Responses. Nat. Cel Biol 20, 888–899. 10.1038/s41556-018-0142-z PMC6186418 30050119

[B112] Tschaharganeh D. F. Chen X. Latzko P. Malz M. Gaida M. M. Felix K. (2013). Yes-associated Protein Up-Regulates Jagged-1 and Activates the Notch Pathway in Human Hepatocellular Carcinoma. Gastroenterology 144, 1530–1542. e12. 10.1053/j.gastro.2013.02.009 23419361 PMC3665638

[B113] Ueyama T. Zhu C. Valenzuela Y. M. Suzow J. G. Stewart A. F. R. (2000). Identification of the Functional Domain in the Transcription Factor RTEF-1 that Mediates α1-Adrenergic Signaling in Hypertrophied Cardiac Myocytes. J. Biol. Chem. 275, 17476–17480. 10.1074/jbc.m001970200 10764782

[B114] Vassilev A. Kaneko K. J. Shu H. Zhao Y. Depamphilis M. L. (2001). TEAD/TEF Transcription Factors Utilize the Activation Domain of YAP65, a Src/Yes-Associated Protein Localized in the Cytoplasm. Genes Dev. 15, 1229–1241. 10.1101/gad.888601 11358867 PMC313800

[B115] Vigneswaran K. Boyd N. H. Oh S.-Y. Lallani S. Boucher A. Neill S. G. (2021). YAP/TAZ Transcriptional Coactivators Create Therapeutic Vulnerability to Verteporfin in EGFR-Mutant Glioblastoma. Clin. Cancer Res. 27, 1553–1569. 10.1158/1078-0432.ccr-20-0018 33172899 PMC8440125

[B116] Wang C. Jeong K. Jiang H. Guo W. Gu C. Lu Y. (2016a). YAP/TAZ Regulates the Insulin Signaling via IRS1/2 in Endometrial Cancer. Am. J. Cancer Res. 6, 996–1010. 27293994 PMC4889715

[B117] Wang C. Zhu X. Feng W. Yu Y. Jeong K. Guo W. (2016b). Verteporfin Inhibits YAP Function through Up-Regulating 14-3-3σ Sequestering YAP in the Cytoplasm. Am. J. Cancer Res. 6, 27–37. 27073720 PMC4759394

[B118] Wang C. Gu C. Jeong K. J. Zhang D. Guo W. Lu Y. (2017). YAP/TAZ-Mediated Upregulation of GAB2 Leads to Increased Sensitivity to Growth Factor-Induced Activation of the PI3K Pathway. Cancer Res. 77, 1637–1648. 10.1158/0008-5472.can-15-3084 28202507 PMC6026015

[B119] Wang C. Nie Z. Zhou Z. Zhang H. Liu R. Wu J. (2015). The Interplay between TEAD4 and KLF5 Promotes Breast Cancer Partially through Inhibiting the Transcription of p27Kip1. Oncotarget 6, 17685–17697. 10.18632/oncotarget.3779 25970772 PMC4627338

[B120] Wang H. Shi Y. Chen C.-H. Wen Y. Zhou Z. Yang C. (2021). KLF5-induced lncRNA IGFL2-AS1 Promotes Basal-like Breast Cancer Cell Growth and Survival by Upregulating the Expression of IGFL1. Cancer Lett. 515, 49–62. 10.1016/j.canlet.2021.04.016 34052325

[B121] Wang J. Shen C. Zhang J. Zhang Y. Liang Z. Niu H. (2021). TEAD4 Is an Immune Regulating-Related Prognostic Biomarker for Bladder Cancer and Possesses Generalization Value in Pan-Cancer. DNA Cel Biol. 40, 798–810. 10.1089/dna.2021.0164 34030484

[B122] Wang W. Li N. Li X. Tran M. K. Han X. Chen J. (2015). Tankyrase Inhibitors Target YAP by Stabilizing Angiomotin Family Proteins. Cel Rep. 13, 524–532. 10.1016/j.celrep.2015.09.014 PMC4618173 26456820

[B123] Wei C. Li X. (2020). The Role of Photoactivated and Non-photoactivated Verteporfin on Tumor. Front. Pharmacol. 11, 557429. 10.3389/fphar.2020.557429 33178014 PMC7593515

[B124] Weiler S. M. E. Pinna F. Wolf T. Lutz T. Geldiyev A. Sticht C. (2017). Induction of Chromosome Instability by Activation of Yes-Associated Protein and Forkhead Box M1 in Liver Cancer. Gastroenterology 152, 2037–2051. 10.1053/j.gastro.2017.02.018 28249813

[B125] Wen X. Wan J. He Q. Wang M. Li S. Jiang M. (2020). p190A Inactivating Mutations Cause Aberrant RhoA Activation and Promote Malignant Transformation via the Hippo-YAP Pathway in Endometrial Cancer. Sig Transduct Target. Ther. 5, 81. 10.1038/s41392-020-0170-6 PMC7250911 32457342

[B126] Wu L. Yang X. (2018). Targeting the Hippo Pathway for Breast Cancer Therapy. Cancers (Basel) 10, 422. 10.3390/cancers10110422 PMC6266939 30400599

[B127] Xia H. Wen J. Zhao W. Gu D. Hu Z. Chen J. (2017). The Prognostic Impacts of TEA Domain (TEAD) Transcription Factor Polymorphisms in Chinese Hepatocellular Carcinoma Patients. Oncotarget 8, 69823–69832. 10.18632/oncotarget.19310 29050244 PMC5642519

[B128] Xia J. Zeng M. Zhu H. Chen X. Weng Z. Li S. (2018). Emerging Role of Hippo Signalling Pathway in Bladder Cancer. J. Cel. Mol. Med. 22, 4–15. 10.1111/jcmm.13293 PMC5742740 28782275

[B129] Xia Y. Chang T. Wang Y. Liu Y. Li W. Li M. (2014a). YAP Promotes Ovarian Cancer Cell Tumorigenesis and Is Indicative of a Poor Prognosis for Ovarian Cancer Patients. PLoS One 9, e91770. 10.1371/journal.pone.0091770 24622501 PMC3951505

[B130] Xia Y. Zhang Y.-L. Yu C. Chang T. Fan H.-Y. (2014b). YAP/TEAD Co-activator Regulated Pluripotency and Chemoresistance in Ovarian Cancer Initiated Cells. PLoS One 9, e109575. 10.1371/journal.pone.0109575 25369529 PMC4219672

[B131] Xu X. Nie J. Lu L. du C. Meng F. Song D. (2021). YAP‐TEAD Up‐regulates IRS2 Expression to Induce and Deteriorate Oesophageal Cancer. J. Cell. Mol. Medi 25, 2584–2595. 10.1111/jcmm.16266 PMC7933937 33570213

[B132] Yagi R. Kohn M. J. Karavanova I. Kaneko K. J. Vullhorst D. Depamphilis M. L. (2007). Transcription Factor TEAD4 Specifies the Trophectoderm Lineage at the Beginning of Mammalian Development. Development 134, 3827–3836. 10.1242/dev.010223 17913785

[B133] Yu F.-X. Meng Z. Plouffe S. W. Guan K.-L. (2015). Hippo Pathway Regulation of Gastrointestinal Tissues. Annu. Rev. Physiol. 77, 201–227. 10.1146/annurev-physiol-021014-071733 25293527

[B134] Yu T. Song J. Zhou H. Wu T. Liang Z. du P. (2021). Nuclear TEAD4 with SIX1 Overexpression Is an Independent Prognostic Marker in the Stage I-III Colorectal Cancer. Cmar Vol. 13, 1581–1589. 10.2147/cmar.s260790 PMC7898202 33628048

[B135] Yuan H.-Y. Lv Y.-J. Chen Y. Li D. Li X. Qu J. (2021). TEAD4 Is a Novel Independent Predictor of Prognosis in LGG Patients with IDH Mutation. Open Life Sci. 16, 323–335. 10.1515/biol-2021-0039 33889755 PMC8042920

[B149] Yuan H. Liu H. Liu Z. Zhu D. Amos C. I. Fang S. (2015). Genetic Variants in Hippo Pathway Genes YAP1, TEAD1 and TEAD4 are Associated With Melanoma-Specific Survival. Int. J. Cancer 137 (3), 638–645. 25628125 10.1002/ijc.29429 PMC4437894

[B136] Zanconato F. Cordenonsi M. Piccolo S. (2016). YAP/TAZ at the Roots of Cancer. Cancer Cell 29, 783–803. 10.1016/j.ccell.2016.05.005 27300434 PMC6186419

[B137] Zanconato F. Forcato M. Battilana G. Azzolin L. Quaranta E. Bodega B. (2015). Genome-wide Association between YAP/TAZ/TEAD and AP-1 at Enhancers Drives Oncogenic Growth. Nat. Cel Biol 17, 1218–1227. 10.1038/ncb3216 PMC6186417 26258633

[B138] Zemke N. R. Gou D. Berk A. J. (2019). Dedifferentiation by Adenovirus E1A Due to Inactivation of Hippo Pathway Effectors YAP and TAZ. Genes Dev. 33, 828–843. 10.1101/gad.324814.119 31171701 PMC6601516

[B139] Zhang B. Wang Q. Ji Y. Zhang X. Xue L. Shi Q. (2022). TEAD4 Overexpression Suppresses Thyroid Cancer Progression and Metastasis *In Vitro* by Modulating Wnt Signaling. J. Biosci. 47, 3. 10.1007/s12038-021-00238-3 34951409

[B140] Zhang H. Geng D. Gao J. Qi Y. Shi Y. Wang Y. (2016). Expression and Significance of Hippo/YAP Signaling in Glioma Progression. Tumor Biol. 37, 15665–15676. 10.1007/s13277-016-5318-1 27718125

[B141] Zhang W. Li J. Wu Y. Ge H. Song Y. Wang D. (2018a). TEAD4 Overexpression Promotes Epithelial-Mesenchymal Transition and Associates with Aggressiveness and Adverse Prognosis in Head Neck Squamous Cell Carcinoma. Cancer Cel Int 18, 178. 10.1186/s12935-018-0675-z PMC6233371 30459528

[B142] Zhang W. Xu J. Li J. Guo T. Jiang D. Feng X. (2018b). The TEA Domain Family Transcription Factor TEAD4 Represses Murine Adipogenesis by Recruiting the Cofactors VGLL4 and CtBP2 into a Transcriptional Complex. J. Biol. Chem. 293, 17119–17134. 10.1074/jbc.ra118.003608 30209132 PMC6222106

[B143] Zhang Z. Lin Z. Zhou Z. Shen H. C. Yan S. F. Mayweg A. V. (2014). Structure-Based Design and Synthesis of Potent Cyclic Peptides Inhibiting the YAP-TEAD Protein-Protein Interaction. ACS Med. Chem. Lett. 5, 993–998. 10.1021/ml500160m 25221655 PMC4160762

[B144] Zheng D. Cao M. Zuo S. Xia X. Zhi C. Lin Y. (2022). RANBP1 Promotes Colorectal Cancer Progression by Regulating Pre-miRNA Nuclear export via a Positive Feedback Loop with YAP. Oncogene 41, 930–942. 10.1038/s41388-021-02036-5 34615998

[B145] Zhou Y. Huang T. Cheng A. S. Yu J. Kang W. To K. F. (2016). The TEAD Family and its Oncogenic Role in Promoting Tumorigenesis. Int. J. Mol. Sci. 17, 138. 10.3390/ijms17010138 PMC4730377 26805820

[B146] Zhou Y. Huang T. Zhang J. Wong C. C. Zhang B. Dong Y. (2017). TEAD1/4 Exerts Oncogenic Role and Is Negatively Regulated by miR-4269 in Gastric Tumorigenesis. Oncogene 36, 6518–6530. 10.1038/onc.2017.257 28759040 PMC5702719

[B147] Zhou Z. Hu T. Xu Z. Lin Z. Zhang Z. Feng T. (2015). Targeting Hippo Pathway by Specific Interruption of YAP‐TEAD Interaction Using Cyclic YAP‐like Peptides. FASEB j. 29, 724–732. 10.1096/fj.14-262980 25384421

[B148] Zhu C. Li L. Zhang Z. Bi M. Wang H. Su W. (2019). A Non-canonical Role of YAP/TEAD Is Required for Activation of Estrogen-Regulated Enhancers in Breast Cancer. Mol. Cel 75, 791–806. e8. 10.1016/j.molcel.2019.06.010 PMC6707877 31303470

